# Carotenoid Cleavage Oxygenases from Microbes and Photosynthetic Organisms: Features and Functions

**DOI:** 10.3390/ijms17111781

**Published:** 2016-10-26

**Authors:** Oussama Ahrazem, Lourdes Gómez-Gómez, María J. Rodrigo, Javier Avalos, María Carmen Limón

**Affiliations:** 1 Instituto Botánico, Departamento de Ciencia y Tecnología Agroforestal y Genética, Facultad de Farmacia, Universidad de Castilla-La Mancha, Campus Universitario s/n, 02071 Albacete, Spain; Oussama.Ahrazem@uclm.es (O.A.); MariaLourdes.Gomez@uclm.es (L.G.-G.); 2 Instituto de Agroquímica y Tecnología de Alimentos (IATA-CSIC), Departamento de Ciencia de los Alimentos, Calle Catedrático Agustín Escardino 7, 46980 Paterna, Spain; mjrodrigo@iata.csic.es; 3 Departamento de Genética, Facultad de Biología, Universidad de Sevilla, Avenida Reina Mercedes 6, 41012 Sevilla, Spain; avalos@us.es

**Keywords:** algae, apocarotenoids, bacteria, carotenoid cleavage dioxygenase, fungi, plants

## Abstract

Apocarotenoids are carotenoid-derived compounds widespread in all major taxonomic groups, where they play important roles in different physiological processes. In addition, apocarotenoids include compounds with high economic value in food and cosmetics industries. Apocarotenoid biosynthesis starts with the action of carotenoid cleavage dioxygenases (CCDs), a family of non-heme iron enzymes that catalyze the oxidative cleavage of carbon–carbon double bonds in carotenoid backbones through a similar molecular mechanism, generating aldehyde or ketone groups in the cleaving ends. From the identification of the first CCD enzyme in plants, an increasing number of CCDs have been identified in many other species, including microorganisms, proving to be a ubiquitously distributed and evolutionarily conserved enzymatic family. This review focuses on CCDs from plants, algae, fungi, and bacteria, describing recent progress in their functions and regulatory mechanisms in relation to the different roles played by the apocarotenoids in these organisms.

## 1. Introduction

Carotenoids are a large group of terpenoid fat-soluble pigments widely distributed in nature. They are abundantly present in plants, where they are masked by the green color of chlorophyll, but they are responsible for the yellow, orange, or reddish colors of many fruits and flowers. They provide color also to many microorganisms and to some animals, including birds, fishes, and crustaceans. Chemically, the carotenoids are C_40_ polyene compounds with a chain of conjugated double bonds, which creates a chromophore that absorbs light in the UV and blue range of the spectrum. Carotenoids are produced by algae and plants as well as by many fungi and bacteria [1,2,3]. Animals are mostly unable to synthesize carotenoids, but an outstanding exception was found in certain aphids, explained by recent carotenoid biosynthetic gene horizontal transfer from fungi [4,5]. Carotenoids exert important biological functions in most living organisms, usually related with their photoprotective and light-absorbing properties. Their functions are especially relevant in plants, algae, cyanobacteria, and anoxygenic prototrophic bacteria [4,6,7], where they are indispensable in photosynthesis because of their light harvesting and protecting roles [8]. Further, carotenoids act as precursors of a vast group of bioactive compounds, the apocarotenoids, with very diverse biological functions [9].

The electron-rich polyene chain of carotenoids makes them susceptible to oxidative breakdown, leading to the generation of unspecific apocarotenoid products by random cleavage, carried out by carotenoid-unrelated enzymes such as lipoxygenases or peroxidases. However, apocarotenoids are usually the result of a biologically active process, resulting from the action of specific carotenoid cleavage dioxygenases (CCDs), frequently referred to as oxygenases (CCOs), a family of non-heme iron-type enzymes that cleave double bonds in the conjugated carbon chain of carotenoids [10]. Although the first carotenoid cleaving activity was reported in animal tissues [11], the first CCD was identified in the plant *Arabidopsis thaliana* [12], initiating the discovery of a large series of CCD enzymes in many other species. CCDs typically catalyze the cleavage of non-aromatic double bonds by dioxygen to form aldehyde or ketone products. Some CCDs act specifically on apocarotenoid substrates, and these enzymes are known as apocarotenoid cleavage oxygenases (ACOs). In addition to carotenogenic organisms, represented by plants, algae, fungi, and bacteria, CCDs are also widespread in animals, using them to cleave carotenoids acquired through the diet.

This review covers the different CCD families identified hitherto in microorganisms and in photosynthetic species. In the microbial sections, the name CCDs will be generically used to include all types of oxygenases, and the nomenclature ACO will be reserved for the apocarotenoid specific oxygenases. In the plant section, we will refer to the CCD1, 2, 4, 7, and 8 enzyme subfamilies. The members of the nine-*cis*-epoxycarotenoid dioxygenases (NCED) subfamily responsible for the specific cleavage of 9-*cis*-epoxycarotenoids and involved in the production of abscisic acid (ABA) are not included in this review since it has been the subject of numerous studies and their activities and functions are well known [12,13,14,15,16,17,18].

## 2. Bacterial Carotenoid Oxygenases

Biological roles of CCDs in bacteria are not well established. In cyanobacteria, apocarotenoids act as photoprotective and accessory pigments in thylakoid membranes. Cyanobacteria are known also to be responsible for undesired flavors in drinking water and fish from aquiculture. They produce different odor compounds such as fermentation products and apocarotenoids, the former of which is also found in microbially produced dairy food. The apocarotenoids include derivatives of β-ionone, which generally exhibit pleasant odors found in many flowers and are widely used by industry. Apocarotenoids are produced from carotenoids by CCDs, the first of which was described in *Microcystis* PPC 7806 [19]. A very different function is found, however, in some archaea and eubacteria, where these enzymes are essential for the biosynthesis of retinal, the chromophore for rhodopsins, or similar pumps [20,21,22]. In fungi, a similar function has been also described (see fungal section).

### 2.1. Structural Studies

**Figure 1:**
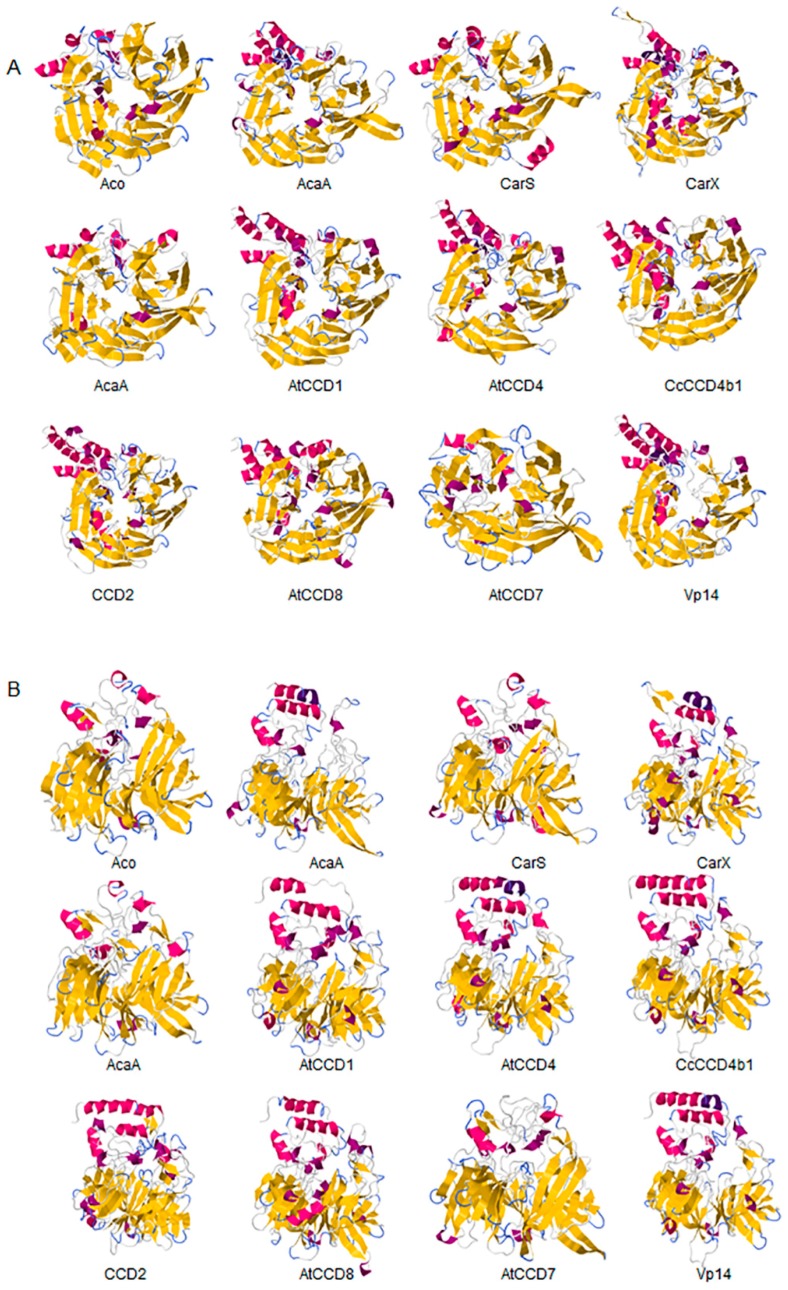

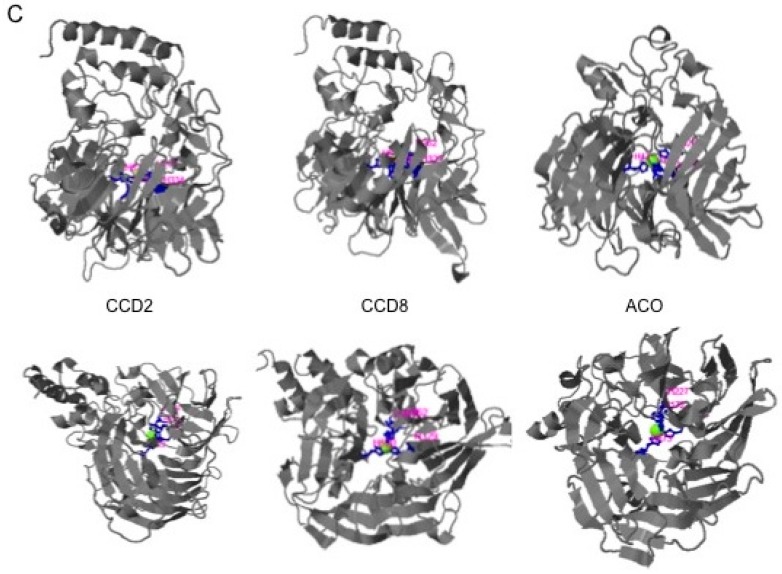
Tridimensional models of 12 carotenoid-cleavage dioxygenases from all the subfamilies included in this review. The VP14 (PBD: 2biwA) structure from maize has been used as a template. (**A**) Side view of CCDs with β-strands shown in yellow, α-helices in magenta, and loops in grey; (**B**) Top view rotated 90° towards the viewer from (**A**); (**C**) Lateral and top views of CCD2, CCD8, and ACO showing Fe^2+^ ion in green and histidines in blue. Accession numbers are: VP14: O24592.2, ACOX, P74334; AtCCD1, O65572; AtCCD7, AEC10494.1; AtCCD8, Q8VY26; AtCCD4: O49675; Cao-2, XP001727958.1; CarS, ADU04395.1; CarX, CAH70723.1; CsCCD2L, ALM23547.1; CcCCD4b1, XP006424046; AcaA, 77754.

The first crystal structure of a CCD was determined for an apocarotenoid cleavage oxygenase (ACO) from *Synechocystis* sp. PCC 6803 [23]. The spatial organization resembles a propeller with seven blades, conserved in all described CCDs and, in fact, a structural signature for all of them. Five blades (I to V) are made of four antiparallel β strands, and two blades (VI and VII) consist of 5 strands (Figure 1) [24].

The active center is located on the top of the enzyme, close to the propeller axis. CCDs contain a Fe^2+^ ion as a cofactor that is indispensable for the cleavage activity. Its putative role is to activate oxygen involved in the enzymatic reaction. The Fe^2+^ is coordinated by four His residues, which are conserved in the CCD family. There is a second coordination center formed by three Glu residues interacting through hydrogen bonds to three of the His residues. The requirement for these amino acids has been demonstrated via mutagenesis [25,26,27].

Another characteristic of CCDs is a large tunnel perpendicular to the propeller axis that enters the protein, passes through the active center, and exits the protein parallel to the propeller axis. The access to the tunnel is important for the entrance of the substrate and is located in a large hydrophobic patch that allows for the localization of the enzyme in the cell membrane. This long tunnel consists of hydrophobic residues (Phe, Val, Leu) and a few aromatic residues (Tyr, Trp, His), forming “van der Waals” forces allowing a correct orientation of the substrate [24]. The hydrophobic and aromatic residues play an important role in isomerase activity, demonstrated through mutagenesis experiments [24].

The propeller-forming β-strands are conserved between ACO (*Synechocystis*) and NOV2 (*Novosphingobium aromaticivorans*). However, there are structural differences in the entrance loop and the dome of both enzymes. The differences in the residues of these structural domains are seemingly involved in their different substrate specificities. In fact, the *Synechocystis* model suggests differences in the substrate requirement compared with the NOV model. In ACO, besides the substrate tunnel, there are two other tunnels made mainly by hydrophobic residues that connect the active site to a hydrophilic mouth. The reaction products are directed to the cytosol through the mouth of the exit tunnel.

### 2.2. Substrate Specificity

Studies on bacterial CCDs usually focused the attention on the purification of different enzymes and the determination of their specificity through their incubation with diverse carotenoid substrates. Frequently, the enzymes exhibit a high specificity, cleaving at a certain position of the polyene chain, while others are less specific. The available information is summarized below.

#### 2.2.1. Apocarotenoid Cleavage Oxygenases (ACO)

ACO enzymes cleave exclusively at C15–C15′ double bonds of apo-β-carotenals. The best-known ACOs are Diox1 from *Synechocystis* sp. PCC 6803 and NosACO, from *Nostoc* sp. PCC 7120 (Table 1). Diox1 cleaves at the C15–C15′ double bond of certain all-*trans*-apocarotenoids, such as apo-β-carotenals, apo-β-carotenols, apo-8′-lycopenal, and apo-lycopenol [28,29]. The enzyme is rather unspecific, since it accepts apo-β-carotenals, apo-β-carotenols, and their 3-hydroxy derivatives, with lengths ranging from C_25_ (12′-apo) to C_35_ (4′-apo), to generate a C_20_ retinal (or 3-hydroxy-retinal) and a dialdehyde product (between C_5_ to C_15_) (Table 1). However, larger substrates, such as β-carotene (C_40_), are not cleaved [29].

NosACO, also named NSC2 [30], is one of the three CCDs of the cyanobacterium *Nostoc* sp. (strain PCC 7120). The three enzymes exhibit different substrate preferences, as expected from cooperative functions among them [31]. In vitro, NosACO cleaves monocyclic or acyclic carotenoids at C15–C15′ double bonds to generate retinal. In vivo, bleaching activity of NosACO was also observed on β-carotene, zeaxanthin, torulene, lycopene, and diapocarotenedial [32,33] (Table 1).

#### 2.2.2. CCDs with Symmetrical Mode of Action

A β-carotene oxygenase from *Microcystis*, not identified yet, is able to carry out two symmetrical cleavages on β-carotene and zeaxanthin at the C7–C8 and C7′–C8′ double bonds [34]. It has been suggested that one molecule of crocetindial and two molecules of β-cyclocitral are released from each molecule of β-carotene [19]. On the other hand, one molecule of crocetindial and two molecules of hydroxyl-β-cyclocitral would be released from a molecule of zeaxanthin.

Carotenoid oxygenases are responsible for the generation of some bacterial aromatic compounds, as those produced by cyanobacterial species of the genera *Calotrix* and *Plectonema*. For instance, *Plectonema notatum* PCC 6306 and *Plectonema* sp. PCC 7410 produced 6-methyl-5-hepten-2-one, β-ionone, 2,6,6-trimethylcyclohexanone, β-cyclocitral, 2-hydroxy-2,6,6-trimethylcyclo-hexan-1-one, 6-methyl-5-hepten-2-ol, dihydroactinidiolide, and β-ionone-5,6-epoxide. Moreover, *Plectonema notatum* PCC 6306 and *Plectonema* sp. PCC 7410 produced cyclogeraniol, 4-oxo-β-ionone, and dihydro-β-ionol. Based on *Microcystis* data, carotenoid oxygenases may be also present in biofilms with *Phormidium* sp., *Rivularia* sp., and *Tolypothrix distorta* [35].

#### 2.2.3. Carotenoid Oxygenases Cleaving at Different Positions

NSC1, also known as NosCCD, is a soluble CCD from *Nostoc* sp. PCC 7120 that shares 44% homology with AtCCD1 from *Arabidopsis thaliana*, but only 26% identity with NSC3, also from *Nostoc* sp. When purified, NSC1 was incubated with β-apo-8′-carotenal in vitro, HPLC chromatograms showed a peak corresponding to 8,10′-apocarotenal, and a second product was identified by GC-MS as the volatile β-ionone [33]. If the incubation was kept for 5 h, the product 8,10′-apocarotenal was not accumulated proportionally to the disappearance of the substrate. Moreover, when the incubation was prolonged to 14 h, the 8,10′-apocarotenal disappeared, and isomers of 8,10′-apocarotenal and additional products resulting from the cleavage of β-apo-8′-carotenal at its C7–C8 and C9′–C10′ bonds, were identified [33]. In any case, the main target were the C9–C10 double bonds and cleavage at other positions releases only minor products that may be a mechanism of eliminating competing species. In summary, in vitro NSC1 cleaves at C9–C10 and C9′–C10′ double bonds in bicyclic carotenoids and at C9–C10 and C7′–C8′ double bonds in monocyclic carotenoids. Interestingly, β,β-carotene cleavage products such as β-ionone, methylheptenone, and geranylacetone are known to inhibit growth of some cyanobacteria [35].

NSC3, also named NosDiox2, cleaves β-apo-8′-carotenals at C13–C14, C13′–C14′, and C15–C15′ double bonds in vitro; in vivo, NSC3 cleaved C_30_ compounds, such as 4,4′-diaponeurosporene at the C13–C14′ double bond and 4,4′-diaponeurosporen-4′-al at the C9′–C10′ double bond. Using C_40_ carotenoids as substrate, NSC3 cleaved torulene at C15–C15′ double bond and seemingly also 3,4,3′,4′-tetrahydrolycopene, but the cleaving site was not identified in that case; however, no products were detected using lycopene [31]. The explanation could be that the fully conjugated double bonds of the carotenoid backbone could facilitate the entry and the binding to the active pocket of the enzyme [23]. In vitro, 4,4′-diaponeurosporene and 4,4′-diaponeurosporen-4′-al were cleaved at more sites than in vivo. Discrepancies were observed in torulene cleavage by NSC3, which might be due to the differences among *Escherichia coli* strains and protein solubilisation [33]. Apocarotenals and apocarotendials were shown to have anticancer effects, suggesting that NSC3 could be used biotechnologically to produce novel bioactive compounds [31].

Several mycobacterial species are known to synthesize carotenoids; however, *Mycobacterium tuberculosis* does not contain carotenogenic genes, which were probably lost during evolution. Nevertheless, two ORFs coding for putative carotenoid cleavage oxygenases, *Rv0654* and *Rv0913c*, were found in the genome of this species. *Rv0654*, named MtCCO from *M. tuberculosis* carotenoid cleavage oxygenase [36], shows a 44% similarity with the Nostoc CCO [30] and contains the four conserved His residues involved in binding the Fe^2+^ cofactor.

Expression of MtCCO in *E. coli* as a GST fusion protein allowed its biochemical characterization in vitro. β-Apo-10′-carotenal was mainly cleaved by this enzyme at the C13–C14 double bond and less frequently at the C15–C15′double bond, while 3-OH-β-apo-11-carotenal was equally cleaved at both double bonds. MtCCO cleaved also C_30_ compounds efficiently, but exhibited only a weak activity on C_25_ compounds and no activity on shorter molecules, indicating that the substrate of this enzyme must have a minimal length of 25 carbons [36]. MtCCO also showed higher affinity for unsubstituted apocarotenoids, but the conversion was faster on those hydroxylated. Additionally, the enzyme cleaved C_40_ carotenoid substrates both symmetrically at the C15–C15′ and asymmetrically at the C13–C14 or C13′–C14′ double bonds, albeit the symmetrical and the asymmetrical cleavages were not equally targeted among the tested substrates (β-carotene, zeaxanthin, and lutein). For instance, there was a preference for a symmetrical cleavage when the β-ionone ring has a 3-OH radical (see zeaxanthin and lutein in Table 2). On the other hand, the acyclic lycopene was not cleaved by MtCCO in vitro, but it was converted to apo-13-lycopenone and apo-15′-lycopenal (acycloretinal) in lycopene-accumulating *E. coli* cells [36].

The enzyme NACOX1 from *Novosphingobium aromaticivoransi* exhibited cleavage activity at the C13–C14 double bound of carotenoids with a β-ionone ring giving β-apo-13-carotenone as resulting product [37].

Blast searches with the *Nostoc* NosCCD sequence in the genomes of the marine proteobacteria *Sphingopyxis alaskensis* and *Plesiocystis pacifica* retrieved two putative CCD genes whose identities with NosCCD ranged from 27% to 38%. After expression in carotenoid-expressing *E. coli*, only one of the two enzymes encoded by the putative CCD genes from both species, named respectively SaCCO and PpCCO, exhibited carotenoid cleavage activity [38]. Purified SaCCO cleaved apo-8′-carotenal in vitro and released apo-12′-carotenal and apo-10′-carotenal (Table 2); however, lycopene was poorly cleaved, and no activity was found against β-carotene or zeaxanthin. On the other hand, PpCCO cleaved zeaxanthin at the C13′–C14′ double bond, producing apo-13′-zeaxanthinone and apo-14′-zeaxanthinal, and at the C11′–C12′ double bond, releasing apo-12′-zeaxanthinal (Table 2).

Similar products were observed after cantaxanthin, astaxanthin, and nostoxanthin were cleaved by PpCCO at the C13′–C14′ position, producing apo-13′-cantaxanthinone and apo-14′-cantaxanthinal (Table 2), apo-13′-astaxanthinone and apo-14′-astaxanthinal (Table 2), and apo-14′-nostoxanthinal (Table 3), respectively. When PpCCO cleaves cantaxanthin and nostoxanthin at their C11′–C12′ bonds, apo-12′-cantaxanthinal (Table 2) and apo-12′-nostoxanthinal (Table 3) are released, respectively.

## 3. Fungal Carotenoid Oxygenases

Carotenoid production is a frequent trait in fungi. Best-known examples are the production of β-carotene in different taxonomic groups, neurosporaxanthin in ascomycetes, and astaxanthin in basidiomycetes. In some fungi, as *Phycomyces blakesleeanus*, *Mucor circinelloides*, *Neurospora crassa*, *Fusarium fujikuroi*, and *Xanthophyllomyces dendrorhous*, the genetic and biochemical basis of their carotenoid production has received considerable attention, and the functions and regulation of all the structural genes have been thoroughly investigated [2]. In addition, some fungi are excellent carotenoid producers, and they have been adopted as biotechnological carotenoid sources [39,40]. In contrast to photosynthetic organisms, in the cases investigated, the presence of carotenoids is dispensable, and the mutants unable to make carotenoids are viable. In some fungal models, such as *N. crassa*, such mutants have been widely used as easily traceable genetic markers.

**Figure 2:**
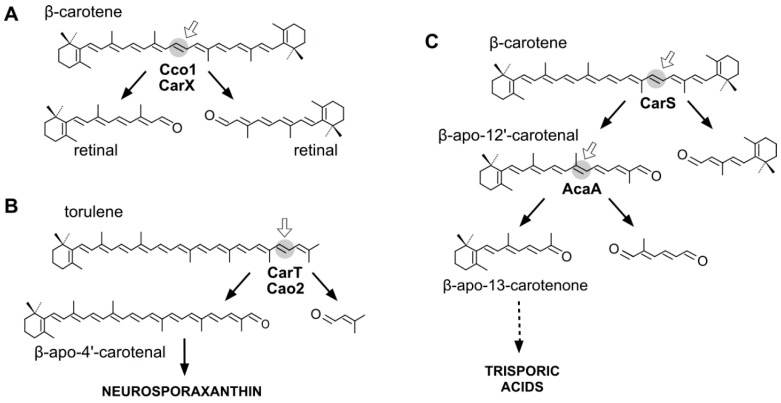
Enzymatic reactions achieved by fungal CCDs. (**A**) Retinal production from β-carotene by the Cco1 (*Ustilago maydis*) and CarX (*F. fujikuroi*) CCDs; (**B**) β-apo-4′-carotenal production from torulene by the CarT (*F. fujikuroi*) and Cao-2 (*N. crassa*) CCDs; (**C**) β-apo-13-carotenone production from β-carotene by the sequential cleavage by the CarS and AcaA CCDs (*P. blakesleeanus*). Cleavage sites are shaded and indicated by an arrow.

In fungi, carotenoid biosynthesis derives from the mevalonate pathway, as indicates the labeling of carotenoids with radioactive mevalonate [41,42]. The biosynthetic pathways are similar to those of photosynthetic species, except that a single desaturase is responsible for all desaturation steps from phytoene, and the cyclase and phytoene synthase activities depend on a single bifunctional gene. Several CCD enzymes have been identified in fungi participating in late steps of their carotenoid pathways. They are related to the production of three different compounds: retinal, neurosporaxanthin, and trisporic acids (Figure 2).

### 3.1. Fungal CCDs Involved in Retinal Production

Rhodopsins are membrane photoreceptors using retinal as a chromophore. The tertiary structure of these proteins is highly conserved and consists of seven transmembrane helices with a lysine residue to which an all-*trans*-retinal chromophore is covalently bound. Rhodopsins are found in all major taxonomic groups, including fungi [43]. Very few fungal rhodopsins have been functionally investigated, among them NOP-1 in *N. crassa* [44], Ops in *Leptosphaeria maculans* [45,46], and CarO [47], and OpsA in *F. fujikuroi* [48]. The participation of retinal in the activity of these proteins has only been demonstrated for NOP-1 [49] and CarO [50].

Animals obtain retinal from β-carotene through its oxidative cleavage by a class of CCD enzymes [51]. A similar CCD, encoded by the gene *carX* [52], is used by *F. fujikuroi* to produce retinal from β-carotene (Figure 2) [53]. The gene *carX* is located in a coregulated cluster with the genes needed to produce β-carotene, *carRA*, and *carB*, and with the rhodopsin gene *carO*. Unexpectedly, despite the biochemical similarities between their carotenoid pathways, no *carX* ortholog has been identified in *N. crassa*. The only possible candidate for such activity in the genome of this fungus, the CCD CAO-1, was not active on carotenoid substrates, but cleaved efficiently the stilbene resveratrol [54]. A retinal-forming CCD enzyme, called Cco1, has also been described in the basidiomycete *Ustilago maydis*, producing minor amounts of β-carotene [55]. In this organism, retinal could be detected in cell extracts of the wild type and strains overexpressing *cco1*, but not in those of null *cco1* mutants, and the in vivo β-carotene levels correlated inversely with the Cco1 activity. Retinal production seems to be particularly relevant in this fungus, as suggested by the occurrence of three genes in its genome for presumptive photoactive rhodopsins [55].

### 3.2. Fungal CCDs Involved in Trisporic Acid Production

Trisporic acids, a class of fungal sexual hormones belonging to the trisporoids family, stand out among the compounds derived from β-carotene for their biological relevance [56,57]. Depending on small variations in their chemical structures, these hormones are distributed in five groups, named A, B, C, D, and E [56,58,59]. The synthesis, investigated in detail in *B. trispora* and other related species, requires the participation of two CCD enzymes. In fact, thorough chemical analyses revealed the generation of an unexpected apocarotenoid complexity in these fungi: *B. trispora* produces at least three groups of β-carotene derivatives—C_18_ trisporoids, C_15_ cyclofarnesoids, and C_7_ methylhexanoids [60]—and the same compounds were found in *P. blakesleeanus* cultures [61]. In the latter case, the origin from β-carotene was confirmed by their absence in a mutant unable to produce β-carotene.

The first CCD enzymes known in fungi were Tsp3 and Tsp4 from *Rhizopus oryzae*, identified through the analysis of its genome and involved in trisporic acid biosynthesis [62]. This discovery led to identification of the *tsp3* ortholog in *B. trispora*, whose connection with trisporic acids was reinforced by its induced expression by sexual interaction and its capacity to cleave β-carotene in a *tsp3*-expressing *E. coli* strain. Further evidence was obtained from the chemical structures of the apocarotenoids produced by *P. blakesleeanus* [63], which were consistent with the cleavage of β-carotene at the C11′–C12′ and C12–C13 double bonds (Figure 2).

The *carS* mutants of *P. blakesleeanus*, which accumulate large amounts of β-carotene, were found to be affected in a CCD enzyme [64], orthologous of Tsp3. The function of the gene, corroborated by the finding of relevant mutations in six different *carS* mutants, was confirmed by the capacity of the CarS protein to generate β-apo-12′-carotenal (Figure 2) in *carS*-expressing *E. coli* cells [65]. The identification of CarS as a CCD was unexpected, since the β-carotene over-accumulation phenotype of *carS* mutants suggested that the gene encoded a regulatory protein. This finding leads to the reinterpretation of the carotene overproduction of the *carS* mutation as a result of a blockage of the pathway or of the lack of a negative-acting apocarotenoid signal, which could be responsible for a formerly proposed feed-back regulation [66]. In support of the blockage hypothesis, the large increase in β-carotene content in these mutants is not sufficiently explained by the minor changes that could be found in the transcript levels of the structural genes *carB* and *carRA* [67]. However, the function of CarS as a CCD enzyme does not discard an additional regulatory role, as suggested by the unexpected albino phenotype of some double *carS* mutants [68]. Moreover, the *carS* mutants exhibit increased enzymatic activities in vitro [42], a result more coherent with a putative regulatory hypothesis.

A second CCD enzyme, AcaA, has been characterized in *P. blakeseleeanus*. AcaA cleaves β-apo-12′-carotenal (C_25_) to generate β-apo-13-carotenone (C_18_) [65]. The available information indicates that CarS and AcaA act sequentially to produce apocarotenoids in this fungus. First, CarS cleaves β-carotene at the C11′–C12′ double bond to generate the C_25_ and C_15_ apocarotenals, and AcaA cleaves the resulting C_25_-product at the C13–C14 double bond afterwards (Figure 2). The resulting C_18_ product is the origin of the trisporic acids and other trisporoids, while the former CarS C_15_ product is used for the synthesis of cyclofarnesoids.

The genome of *P. blakesleeanus* contains three additional genes for presumptive CCDs. Only one of them contains the expected histidine residues to support CCD cleavage activity, but its function has not been investigated. The genes and enzymes for later steps of trisporoid metabolism are currently under study, but they are presumably CCD-unrelated enzymes: they include at least a 4-dihydromethyltrisporate dehydrogenase [69,70] and a 4-dihydrotrisporin-dehydrogenase [71], both identified in the *P. blasleeanus* relative *Mucor mucedo*.

### 3.3. Fungal CCDs Involved in Neurosporaxanthin Production

Neurosporaxanthin is a C_35_ carotenoid derived from the oxidative cleavage of its C_40_ precursor torulene. The first enzymatic reactions in the neurosporaxanthin biosynthetic pathway are similar to those for β-carotene production in other fungi, but in this case five desaturations instead of four and only one cyclization are introduced into the C_40_ carotene skeleton, leading to torulene (Figure 2). The analysis of the proteomes of *N. crassa* and *F. fujikuroi* and the study of mutants blocked in different steps of the pathway led to the identification of the enzymes involved in the late oxidative reactions. The genomes of both species contain two CCD-encoding genes. One of them, called *carT* in *F. fujikuroi*, was found to catalyze the cleavage of torulene to produce β-apo-12′-carotenal. This function was corroborated by the finding of a mutated *carT* allele in a reddish torulene -accumulating mutant and the ability of the wild-type *carT* allele to restore neurosporaxanthin production when it was introduced in the mutant [53,72]. CarT activity was also confirmed by targeted mutation in *Gibberella zeae*, a teleomorph of *Fusarium graminearum* [73]. The same function was achieved in *N. crassa* by its ortholog, *cao-2*, as demonstrated the accumulation of torulene in a mutant for this gene and the finding of *cao-2* mutations in two torulene-producing mutants of this fungus [74].

As found for β-carotene production in Mucorales, the synthesis of neurosporaxanthin is induced by the light in *N. crassa* [75] and *Fusarium* sp. [76]. This photoresponse is achieved through an outstanding increase in mRNA levels for most of the structural genes, including *cao-2* [74] in *N. crassa*, and *carT* [72] in *F. fujikuroi*. Moreover, the expression of *carT* is enhanced in carotenoid-overproducing mutants.

The CarT and CAO-2 enzymes, catalyzing the asymmetrical cleavage of torulene at its acyclic end to remove a C_5_ segment, represent a novel CCD subgroup. The reaction is highly specific, as shown by the incapacity of CAO-2 to cleave the torulene precursor γ-carotene, indicating the need for five desaturations in the substrate molecule. Subsequently, the product β-apo-12′-carotenal is converted to neurosporaxanthin by the aldehyde dehydrogenase YLO-1 in *N. crassa* [77] and by its ortholog CarD in *F. fujikuroi* [78].

## 4. Plant CCDs

The oxidative cleavage of carotenoids in plants leads to the production of a range of apocarotenoids compounds that serve critical functions including photoprotection, photosynthesis, pigmentation, and signaling [79,80]. Plant CCDs constitute the most abundant group identified so far. These CCDs have been classified into two large families based on whether they are involved or not in the production of abscisic acid (ABA), a hormone involved in drought stress responses and in bad and seed dormancy [81]. Those involved in ABA production are the nine-*cis*-epoxy-carotenoid-dioxygenases (NCEDs), which cleave 9-*cis*-violaxanthin and 9-*cis*-neoxanthin to xanthoxin, the precursor of ABA. In fact, the first CCD cloned from any organism was the Vp14 gene from maize that catalyzed the first committed step in ABA biosynthesis [81]. After the identification of Vp14, other CCD enzymes were isolated, including β-carotene oxygenase (BCO) in mammals involved in the synthesis of vitamin A, CCDs from microorganisms (mentioned in former sections), and other plant CCDs. In plants, five CCD groups have been identified so far–CCD1, CCD2, CCD4, CCD7, and CCD8. However, transcriptome and large-scale genomic sequencing projects in many different plant species are allowing the identification of new species-specific CCDs with yet unknown functions. In fact, biochemical studies have been reported for a limited number of plant CCDs, and the roles they play in different organisms are not fully understood.

### 4.1. CCD1 and CCD2 Subfamilies

**Figure 3:**
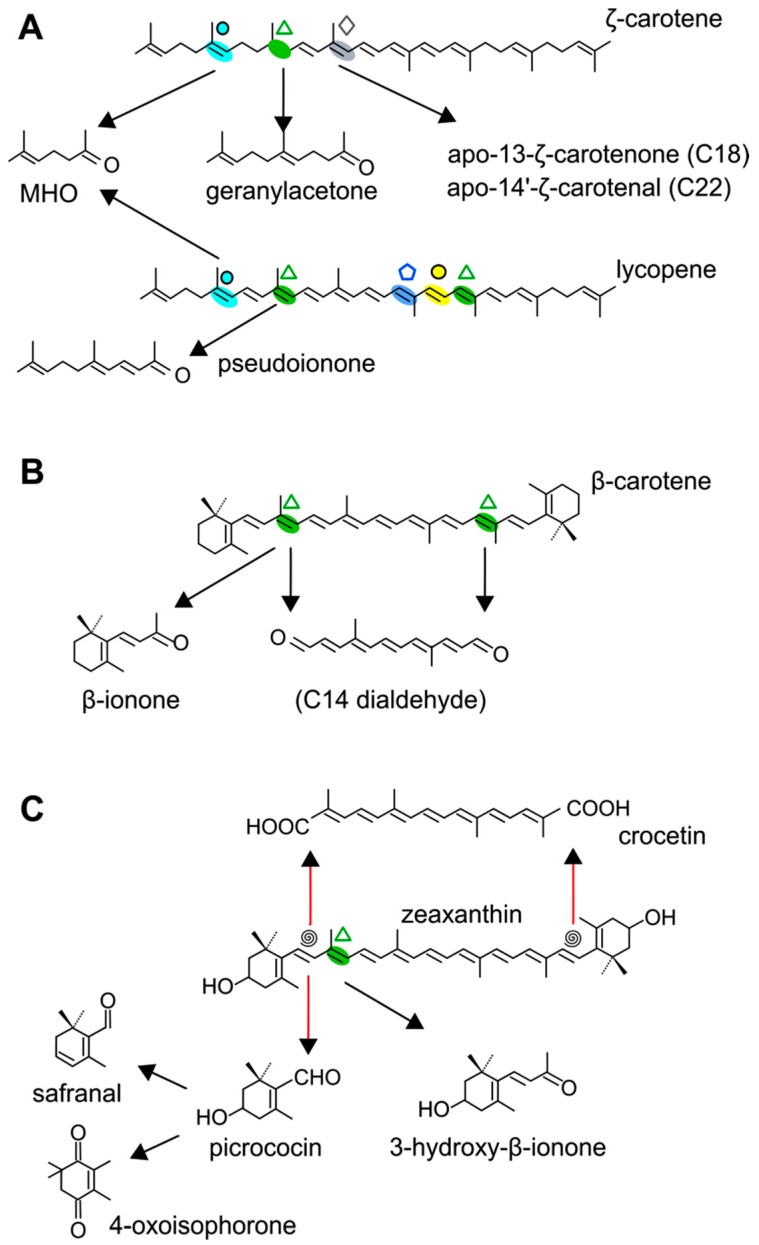
Activity of plant CCD1 and CCD2 subfamilies, showing their divergence in substrate specificity and cleavage sites on the following substrates: (**A**) ζ-carotene and lycopene; (**B**) β-carotene; and (**C**) zeaxantine. Symbols indicate double bonds where oxygenases cleave. 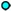: C5–C6; 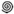: C7–C8; 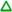: C9–C10 and C9′–C10′; 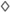: C13–C14; 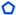: C13′–C14′; 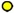: C11′–C12′.

AtCCD1 from *A. thaliana* was the first carotenoid cleavage dioxygenase isolated not involved in ABA biosynthesis (Figure 1) [82]. The first cleavage activity reported for CCD1 enzymes was on the C9–C10 (C9′–C10′) double bonds in C_40_ carotenoids, producing a colored C_14_ dialdehyde and two scent C_13_ products [82]. Homologues of this enzyme have been identified in many other plant species [83,84,85,86,87,88,89,90,91], and the studies performed on these CCD1 enzymes suggested additional cleavage activities on acyclic, monocyclic, and bicyclic carotenoids, such as ζ-carotene, lycopene, phytofluene, β-carotene, δ-carotene, zeaxanthin, lutein, and violaxanthin, and on different apocarotenoids, catalyzing the symmetric cleavage at C9–C10 (C9′–C10′), C5–C6 (C5′–C6′), C7–C8 (C7′–C8′), and C13–C14 (C13′–C14′) (Figure 3) [85,92,93,94,95]. The enzyme cleaved symmetrically at C9–C10 (C9′–C10′) of acyclic and cyclic trans-carotenoids and did not cleave adjacently to a *-cis* double bond or an allenic bond found in some carotenoids [82]. Therefore, an asymmetric cleavage is observed in such cases. The C5–C6 or C5′–C6′ activity of CCD1 enzymes on lycopene (or both), leading to the formation of the C_8_ ketone 6-methyl-5-hepten-2-one (MHO), was reported for the first time in tomato, maize, and *A. thaliana* [92] and was later detected in *Cucumis melo* [84], *Rosa damascena* [96], *Oryza sativa* [97], and *Vitis vinifera* [98]. The cleavage of C7–C8 and C7′–C8′ double bonds of linear and monocyclic carotenoids constitutes a novel recognition site for the CCD1 plant subfamily. So far only detected by in vitro assays for the rice CCD1 enzyme [85], this activity allowed for the formation of C_10_-aldehyde geranial, suggesting an alternative pathway for the geranial formation in plants.

#### 4.1.1. Mode of Action and Functional Implications

Initially, the CCD1 enzymes were suggested to be involved in the biosynthesis of apocarotenoid volatiles, such as geranylacetone, pseudoionone, and β-ionone [79], which possess an extremely low threshold for human perception [99]. Lowering of CCD1 expression in tomato or petunia led to significant reductions in the emission rates of β-ionone and geranylacetone, but did not lead to significant changes in the carotenoid concentration [90], suggesting additional roles for the CCD1 enzymes. In addition, the expression of CCD1 during tomato, strawberry, melon, and grape development, or in saffron flowers, did not mirror the emission of apocarotenoid volatiles [83,84,86,89,90]. Furthermore, experiments conducted to identify *loci* affecting volatile composition in tomato did not reveal the implication of CCD1 in the emissions of apocarotenoid volatiles [100]. These are produced from C_40_ carotenoids localized in plastids, and the CCD1 enzymes lack plastid targeting signals and are cytoplasmatic [87,101], suggesting that CCD1 enzymes mainly act in planta as scavengers of carotenoid degradation products of different chain lengths rather than primary cleavers of intact carotenoids [80,85,93,94,98,102].

The key roles of carotenoids in photosynthesis, photomorphogenesis, and plant development suggest that their biosynthesis and degradation is coordinately regulated with processes such as plastid biogenesis, flowering, and fruit development [103]. In addition, the link of carotenoid biosynthesis with those of gibberellin, ABA, and strigolactone phytohormones implies that changes in the composition or content of carotenoids might bring about physiological or biochemical shiftings in plants [4]. In 2008, a role of CCD1 in the production of strigolactones (SLs) during the arbuscular mycorrhiza symbiosis in roots was described [94]. CCD1 cleaves the C_27_ apocarotenoid derivatives produced by the activity of CCD7, which is also involved in the biosynthesis of SLs [104], producing C_13_ α-ionol and C_14_ mycorradicin, which are indicators of a well-established and functional symbiosis [105], reducing SL production and avoiding over-colonization [106].

CCD1 could be also important in plant stress responses. Enhanced CCD1 expression during berry development has been associated with the increased osmotic stress that occurs during ripening, resulting in leaky membranes and concomitant chloroplast degradation [98]. In addition, CCD1 expression is induced during leaf senescence [89,107], suggesting that the enzyme-mediated degradation of carotenoids, apocarotenoids, or both is catalyzed by cytosolic CCD1 enzymes. Furthermore, plant–insect interactions, extreme temperatures, high irradiance, or ultraviolet (UV) stress induce the production of β-ionone and β-cyclocitral among other apocarotenoid volatiles [108,109]. CCD1 expression has been detected in all tested tissues in different plant species [89,90,91,95,98,110,111], but its expression is stimulated by abiotic stress or ABA treatment [112,113].

#### 4.1.2. CCD1 Gene Family and Genomic Organization

The CCD1 family is present in some plant species as a multigene family (Table S1), and studies on maize and tomato CCD1 enzymes have suggested varying expression profiles and different specificities in their activity towards different substrates and in their double bond preferences [90,92,93,102,114]. In most of the analyzed species, there are two genes encoding for CCD1 enzymes, and in many cases the genes are present in tandem in the same chromosome (Table S1). The identities among the paralogous *CCD1* genes range between 90% and 99%. All the *CCD1* genes show several introns in their sequence [115], these positions being conserved [115]. Moreover, the presence of such introns allows for the generation of different splice variants (Table S1). These variants may differ as well in the untranslated regions, with implications in transcript stability, localization, translation, or a combination thereof. It is likely that the truncated proteins from the *CCD1* mRNA isoforms may act as dominant-negative regulators. In fact, several reports provide evidences of a dominant regulatory role for truncated proteins generated by splice variants in plants [116]. AtCCD1 has been suggested to act as a dimer [82], and the presence of these variants could be part of a regulatory mechanism for CCD1 activity.

The phylogenetic analysis of the CCD1 proteins reveals several main clusters that fit with the phylogenetic relationships in embryophytes (Figure S1). In the angiosperm group, there are three sub-clusters corresponding to monocots, dicots (excluding crucifers), and crucifers. The latter sub-cluster of *Brassicaceae* species is more related to the monocot group than to the dicot group, suggesting a specific function for CCD1 in these plants.

#### 4.1.3. Mode of Action of CCD2

Recently, a close CCD subfamily related to the CCD1 family has been identified in *Crocus* species [117,118]. This subfamily, named as CCD2 (Figure 1), is involved in the formation of the apocarotenoid crocetin, which accumulates in stigma tissue at high levels [119]. Crocetin is derived by bio-oxidative cleavage of zeaxanthin by a C7–C8 and C7′–C8′ cleavage reaction catalyzed by CsCCD2 [117]. The expression of *CsCCD2* is restricted to the stigma in saffron, and it mirrors the levels of crocetin in this tissue during its development [89,117]. The same behavior is observed for CaCCD2, a CCD2 homologue isolated from the spring crocus *C. ancyrensis* [118]. Both CCD2 enzymes are plastidic, a major difference with the CCD1 subfamily [118]. No other CCD2 homologues have been identified in other organisms, presumably due to the uncommon ability to synthesize crocetin in plants [120] or bacteria [121]. Besides zeaxanthin, CCD2 is able to recognize and cleave lutein and 3-OH-β-apocarotenals at the C7–C8 position, but it does not cleave β-carotene, lycopene, or β-cryptoxanthin [117].

#### 4.1.4. Structure of CCD1 and CCD2 Enzymes

Based on the crystal structure of VP14 [122], structural models have been built for CCD1 and CCD2. VP14 is a globular protein that showed a characteristic hydrophobic patch formed by the antiparallel α-helices presumably involved in membrane interaction [28]. CCD1 and CCD2 enzymes display from 58% to 39% overall identity with VP14. Specially conserved are the structural elements of the CCD enzymes. Three regions have been identified in VP14, implicated in the recognition of the bond to be cleaved [122]. The first one is Val478, which is a substitute for a Phe residue in CCD1 and CCD2 enzymes. This substitution is present in many others CCDs. The second region is a loop formed by residues 499 to 503 in VP14. This loop is not present in CCD1 and CCD2 enzymes, in which conserved Leu170 in Vp14 was replaced by Trp. In Vp14, the loop and the leucine residue form a pocket where the second ring of the carotenoid accommodates in such a way that the C11–C12 double bond of the substrate should be close to the Fe^2+^. The third region, located in the active center, comprises three Phe residues in Vp14 (Phe171, Phe411, and Phe589) and is conserved in all NCED and CCD1 enzymes; however, in CCD2, only one Phe residue is conserved.

#### 4.1.5. Regulation of CCD1 and CCD2 Enzymes

As stated before, CsCCD2 expression is regulated during the development of the stigma and the enzyme is localized in chromoplasts [123] where catalyzes crocetin biosynthesis. Crocetin is oxidated and glucosylated thereafter [124], generating crocins that accumulate in the vacuole. The mechanisms involved in CsCCD2 expression and activity have recently been elucidated [123]. In this study, the authors showed that high temperatures repressed CsCCD2 expression, while low temperatures have an opposite effect by upregulating its expression. In saffron plants, the highest expression levels of CsCCD2 are related to the lowest temperatures. Several classes of *cis*-regulatory elements were identified in the promoter of CsCCD2. The regulation of carotenoid biosynthesis by light and dark conditions has been investigated in red pepper [125], in tomato leaves [126], and in citrus, where the expression levels of the carotenogenic genes are affected by circadian rhythms [127,128]. Additionally, a new mechanism of regulation by alternative splicing has been observed for *CsCCD2*, [123]. The obtained data agree with the predicted function of alternative splicing in genes with a circadian clock expression pattern, where oscillations of protein expression levels require rapid adjustments in mRNA levels over the course of a day [129].

### 4.2. The CCD4 Subfamily

The CCD4 subfamily is present in all flowering plants, usually composed by several members (Figure 1) [115,130]. Their functions have been directly linked to coloration and production of aroma volatiles in flower and fruit tissues [89,98,131,132,133]. More recently, CCD4 activity has been reported in seeds, leaves, and roots associated with carotenoid turnover [107,134] or with the production of novel apocarotenoid-derived signals [135]. The first member of this subfamily was identified in *A. thaliana* [18]; however, its role in carotenoid catabolism was initially described for CCD4a from *Chrysantemum morifolium,* whose activity is responsible for the absence of carotenoid accumulation in petals of white flowers [136].

All CCD4 proteins contain a plastid target peptide; moreover, suborganellar studies in different plant species localized these enzymes in the plastoglobuli together with other carotenoid biosynthetic enzymes, which allows direct access to their substrates [131,137,138,139,140,141]. In both chloro- and chromoplasts, CCD4 is one of the major components of plastoglobuli proteomes [137,140], indicating a role for this enzyme in the regulation of carotenoid metabolism in plastid types with marked differences in functionality and carotenoid composition [142]. Moreover, CCD4 protein level is strongly reduced in Arabidopsis mutants defective in the ABC1K1 and ABC1K3 plastoglobuli kinases, suggesting phosphorylation as a post-transcriptional modification necessary for CCD4 protein stability and supporting main activity and localization in plastoglobuli [143].

#### 4.2.1. Enzyme Structure and Functional Implications

As found for other CCDs, molecular modeling of CCD4 enzymes from the structure of the 9-*cis*-epoxycarotenoid dioxygenase from maize (VP14/NCED) revealed a well-conserved structure among the CCD family [130,133]. The analyzed CCD4s display two main functional domains: the helical domain composed of two antiparallel α-helices, which might penetrate in the hydrophobic core of the plastoglobule where carotenoids accumulate [130,138], and the β-propeller structure that forms a long central tunnel, where Fe^2+^ is coordinated with four conserved His in the active center [130,133]. However, other critical amino acids for NCED activity are not always conserved among all CCD4 members [130,133]. As was the case in CCD1 and CCD2 enzymes, a large number of CCD4 proteins display a Phe residue instead of the conserved Val478 found in NCED, with the exception of CCD4 from alfalfa (CCD4a and b), grape (CCD4e), potato (CCD4b), poplar (CCD4c), and citrus (CCD4b1), where it is substituted by Leu; however, in citrus CCD4c, Val478 is replaced by Met. Other critical residues in VP14 are three Phe at positions 171, 411, and 589, from which only Phe589 is conserved in all CCD4, while Phe411 is highly variable, and Phe171, which also takes part of the motif FDG, is partially conserved and usually substituted by Leu. Another important region for VP14 activity is a loop located on the back side of the substrate pocket, involving residues 499–503 and Leu170. In contrast with CCD1 and CCD2, residues in this loop are highly conserved in the CCD4 subfamily; however, the citrus CCD4b1 is a relevant exception, with the Glu499 and Pro500 reported in VP14 substituted by Lys and Glu, respectively. The Leu170 in VP14 is also well conserved in all CCD4s; however, in CCD4b1, CCD4b, and CCD4e from citrus, potato, and poplar, it is replaced by Phe and in citrus CCD4c by Pro. Although it is difficult to determine exactly the impact of these residue changes in the enzyme activity, it is likely that they may modulate the carotenoid substrate specificity, the cleavage position, or both. Interestingly, functional assays performed with citrus CCD4b1, which accumulates several non-conservative amino acid substitutions, show an unusual cleavage position, having as preferential substrates the hydroxylated β-ionone ring of the xanthophylls. Future studies on comparative 3D modeling of the different members of this subfamily will provide clues as to substrate specificity or cleavage position in carotenoid backbone, which may help to understand their role in vivo.

#### 4.2.2. Gene Family and Genomic Organization

The number of genes for CCD4 enzymes is very variable among different species, with at least two genes identified in most plants [115,144]. The presence of one single *CCD4* gene has been reported only in a limited number of plant species with an available genome sequence, such as *A. thaliana*, *Carica papaya* (papaya), *Prunus persica* (peach), and *Sorghum bicolor* (sorghum) [115,130,144]. By contrast, the largest number of CCD4 genes has been found in *Citrus* sp. (a, b1, b2, c and d), *Populus trichocarpa* (a–e), and *Chrysanthemum morifolium* (a-1–a-4 and b), with five members and with at least one of them being a putative pseudogene [115,133,145,146]. Four members have been identified in *Vitis vinifera* (grapevine) (a, b, c, and e) or *Brassica napus* (rapeseed) (A1, A8, C1, and C3), while three are present in *Theobroma cacao* (cacao) [98,130]. Another common genomic feature of *CCD4* genes is the low presence of introns, most of them being intron-less or containing a single intron [115,144]. Moreover, intron size and position are quite variable among different species. A comprehensive comparative analysis of the genomic structure of *CCD4* from diverse plants showed that introns are located in three different sites and only those located at the 3′ end of the gene maintain a conserved position [115]. Exceptionally, only a few *CCD4* genes contain more than one intron, such as *Osmathus fragans CCD4* and *Solanum lycopersicum CCD4a* and *b*, each with two introns, *Citrus* sp. *CCD4d* with two or four introns, and an uncharacterized *CCD4* from apple (*Malus domestica*) with 85% identity at the protein level to apple *CCD4a* [96] with six introns.

The degree of protein sequence identity among CCD4 members is quite variable, ranging from 30% to 98%, with typical values around 50%–70% [115,130,133,147,148]. In multigene CCD4 families, a high degree of homology (85%–98% identity at the protein level) is usually found between the different members, frequently located in tandem on the same chromosome (i.e., tomato *CCD4a* and *b*, citrus *CCD4b1* and *b2*, chrysamthemum *CCD4a-1* to *a-4*, or *Populus CCD4c* to *e*) [115,133]. As expected, the highest variability is found at the plastid target peptide sequences, while a higher conservation is found in the motifs described as essential for CCD activity [6,28,149]. The moderate conservation within the different CCD4 members of the same family (i.e., citrus CCD4b1 versus CCD4c or grapevine CCD4a/b versus CCD4c/e), and the location in different clusters in the phylogenetic analysis (Figure S2) suggest a divergent evolution and functionality of these members.

#### 4.2.3. CCD4 Activity: Carotenoid Substrates and Cleavage Products

In contrast to other CCD subfamilies, such as NCED or CCD7/8, functional assays of CCD4 indicate that the members are rather heterogeneous in respect of carotenoid substrates and cleavage positions (Table 4). The functional characterization of CCD4 has been an active field of research in the last decade by following three basic strategies: (i) in vivo assays of CCD4-defective plant mutants or CCD4-overexpressing or repressing transgenic plants; (ii) in vivo assays of bacteria over-accumulating different carotenoids and co-expressing CCD4; and (iii) in vitro enzymatic assays performed with recombinant CCD4. Table 4 summarizes the information currently available on CCD4 enzyme activity.

The first comparative study of substrate preferences and cleavage positions of CCD4 enzymes was obtained from in vitro and in vivo assays performed with five CCD4s isolated from *Arabidopsis*, rose (*R. damascene*), osmanthus (*O. fragans*), apple (*M. domestica*), and chrysanthemum (*C. morifolium*) [96]. Most of these CCD4 cleaved β-carotene at C9–C10 and C9′–C10′ positions, rendering C_13_ β-ionone, while no activity was detected on hydroxylated xanthophylls [96]. Further research using *Arabidopsis ccd4* mutants or carotenoid over-accumulating lines revealed that epoxy-xanthophylls (mainly violaxanthin) are likely the substrates in *Arabidopsis* leaves [107,134] to render C_13_ apocarotenoids (cleavage at C9–C10 or C9′–C10′ double bonds) that undergo further glycosylation [134]. Interestingly, changes in carotenoid composition and apocarotenoid profile in *ccd4 Arabidopsis* mutants, during seed maturation or in roots of hyperaccumulating β-carotene plants, suggest that CCD4 also accepts β-carotene as substrate [107,134]. One of the two CCD4s from potato, in this work designated as CCD4a (Figure S2), was functionally characterized. Analysis of the carotenoid profile in tubers from RNAi CCD4 potato lines suggested violaxanthin as the main substrate [150]. However, in-depth biochemical characterization of potato CCD4a using in vitro and in vivo assays points to β-carotene as its main substrate, cleaved at C9–C10 or C9′–C10′ positions [148]. In saffron, in vivo assays showed that CCD4a and b cleave β-carotene and zeaxanthin most likely at the C9–C10 and C9′–C10′ double bonds, while CCD4c cleaves other xanthophylls such as lutein [89,130,151]. In vivo assays of grapevine CCD4a and b indicate that these enzymes, in contrast to all CCD4s characterized so far, do not cleave cyclic carotenoids, with the exception of β-carotene, cleaving linear carotenes neurosporene, lycopene, and β-carotene—only CCD4b preferentially at C9–C10 and C9′–10′ positions [98]. The carotenoid profile from rape (*Brassica napus*) flowers suggests that the member CCD4_C3 cleaves α-carotene or δ-carotene at the C9–C10 position to render α-ionone [152]. Interestingly, in vitro and in vivo assays show that *Citrus* sp. CCD4b1/CCD4 accepts β-ring hydroxylated xanthophylls as preferential substrates, but also accepts β-carotene or α-carotene. In contrast to the other plant CCD4s, citrus CCD4b1 and CCD4 cleaves the carotenoid backbone at C7–C8 or C7′–C8′ double bonds, resembling CCD2 activity, but only at one side of the molecule [131,133].

In summary, in vitro and in vivo assays for most of the CCD4 enzymes point to β-carotene and a C9–C10 or C9′–C10′ double bond cleavage as a favorite substrate and positions, respectively, although some relevant exceptions, such as citrus or grape CCD4, have been identified regarding the substrate or cleavage site. In particular, the exclusive cleavage activity reported for citrus CCD4b1 and CCD4, together with its restricted expression pattern, may indicate that this enzyme belongs to a novel class of CCD more functionally related to the recently characterized CCD2 from saffron [117,118]. In planta data, derived from the analysis of apocarotenoid profiles, the alteration in carotenoid complement in *ccd4* mutants and overexpressed or repressed CCD4 plants, or both, point to xanthophylls as the preferential substrates in vivo [130,133,134,145]. Nevertheless, in some species and tissues, such as potato tubers, chrysanthemum petals, and peach fruit, β-carotene cannot be ruled out as the primary target since alterations in the xanthophylls composition in defective or overexpressing CCD4 plants can be explained by a reduction or increase in β-carotene content as the precursor of β-xanthophylls [136,150,153].

These differences in CCD4 substrate preferences may also reflect the differences in substrate availability or accessibility depending on the types of assay. In the in vitro and in vivo assays, the enzyme has direct access to the potential substrates; this clearly differs from the in planta situation, where carotenoids are integrated in the suborganellar structures of the plastids, and CCD4 is preferentially associated with plastoglobuli, where xanthophylls are synthesized and accumulated. The CCD4 activity on linear carotenes is usually very weak either in vivo or in vitro, with the exception of grapevine CCD4 [98]. Recently, the phenotypic characteristics of the Arabidopsis double mutant *clb5 ccd4* (defective in ZDS and CCD4) suggests that, under this particular scenario of hyper-accumulation of linear upstream carotenes, CCD4 generates an apocarotenoid derived from phytofluene, ζ-carotene, or both, indicating an activity on the backbone of these carotenes [135]. None of the CCD4 characterized so far catalyzes the cleavage of phytoene, in agreement with the hypothesis that a double bond in the carotenoid structure must be adjacent to the cleaved double bond [92,98].

These differences in CCD4 substrate preferences may also reflect the differences in substrate availability or accessibility depending on the types of assay. In the in vitro and in vivo assays, the enzyme has direct access to the potential substrates; this clearly differs from the in planta situation, where carotenoids are integrated in the suborganellar structures of the plastids, and CCD4 is preferentially associated with plastoglobuli, where xanthophylls are synthesized and accumulated. The CCD4 activity on linear carotenes is usually very weak either in vivo or in vitro, with the exception of grapevine CCD4 [98]. Recently, the phenotypic characteristics of the *Arabidopsis* double mutant *clb5 ccd4* (defective in ZDS and CCD4) suggests that, under this particular scenario of hyper-accumulation of linear upstream carotenes, CCD4 generates an apocarotenoid derived from phytofluene, ζ-carotene, or both, indicating an activity on the backbone of these carotenes [135]. None of the CCD4 characterized so far catalyzes the cleavage of phytoene, in agreement with the hypothesis that a double bond in the carotenoid structure must be adjacent to the cleaved double bond [92,98]. In summary, in vitro and in vivo assays for most of the CCD4 enzymes point to β-carotene and C9–C10 or C9′–C10′ double bond cleavage as the favorite substrate and positions, respectively, although some relevant exceptions, such as citrus or grape CCD4, have been identified regarding the substrate or cleavage site. In particular, the exclusive cleavage activity reported for citrus CCD4b1 and CCD4, together with its restricted expression pattern, may indicate that this enzyme belongs to a novel class of CCD more functionally related to the recently characterized CCD2 from saffron [117,118]. In planta data, derived from the analysis of apocarotenoid profiles, the alteration in carotenoid complement in *ccd4* mutants and overexpressed/repressed CCD4 plants, or both, point to xanthophylls as the preferential substrates in vivo [130,133,134,145]. Nevertheless, in some species and tissues such as potato tubers, chrysanthemum petals, or peach fruit, β-carotene cannot be ruled out as the primary target since alterations in the xanthophylls composition in defective or overexpressing CCD4 plants can be explained by a reduction or increase in β-carotene content as the precursor of β-xanthophylls [136,150,153]. These differences in CCD4 substrate preferences may also reflect the differences in substrate availability or accessibility depending on the type of assay. In the in vitro and in vivo assays, the enzyme has direct access to the potential substrates; this clearly differs from the in planta situation, where carotenoids are integrated in the suborganellar structures of the plastids, and CCD4 is preferentially associated with plastoglobuli, where xanthophylls are synthesized and accumulated. The CCD4 activity on linear carotenes is usually very weak either in vivo or in vitro, with the exception of grapevine CCD4 [98]. Recently, the phenotypic characteristics of the Arabidopsis double mutant *clb5 ccd4* (defective in ZDS and CCD4) suggests that, under this particular scenario of hyper-accumulation of linear upstream carotenes, CCD4 generates an apocarotenoid derived from phytofluene, ζ-carotene, or both, indicating an activity on the backbone of these carotenes [135]. None of the CCD4 characterized so far catalyzes the cleavage of phytoene, in agreement with the hypothesis that a double bond in the carotenoid structure must be adjacent to the cleaved double bond [92,98].

#### 4.2.4. Expression Pattern in Plant Tissues

*CCD4* genes are predominantly expressed in flower and fruit tissues, suggesting specific roles in these organs [130,147]. Usually at least one CCD4 member is highly or exclusively expressed in flowers, and its function has been related to the production of nor-isoprenoid aroma volatiles to attract pollinators [96,133,146,148,154,155]. In chrysantemum, *Osmanthus*, and cabbage, the expression of a specific *CCD4* gene in petals is responsible for carotenoid degradation and a lack of flower coloration [136,145,152]. In chrysanthemum, CCD4a is represented by a small multigene family, and the white to yellow color gradation in petals among different cultivars can be explained by different combinations and levels of expression of specific CCD4a genes [145]. In the flower petals of lily (*Lilium brownii* var. colchesteri), *CCD4* transcript levels are associated with a loss of carotenoid content during anthesis, resulting in flower whitening [156]. In saffron, the expression of *CCD4a/b* correlates with the production of the nor-isoprenoid β-ionone in flower tissues, while *CCD4c* is restricted to stigmas and shows a highly regulated expression pattern during flower development [89,130]. In other species, such as sweet orange, tomato, and potato, at least one *CCD4* gene is expressed in flowers, but their involvement in carotenoid degradation has not been elucidated [133,148,150]. Expression of *CCD4* genes has also been reported in many fruits and its role has been associated with the biosynthesis of nor-isoprenoid volatiles or apocarotenoids pigments, which may attract animals to facilitate seed dispersion. In grape, *CCD4a* and *b* genes are expressed in the berry, but only *CCD4b* shows a fruit-specific expression profile, highly upregulated during ripening, linking its activity to nor-isoprenoid volatiles production [98]. In summer squash (*Cucurbita pepo*) varieties with white, green, and yellow-orange coloration, the expression of two *CCD4* genes (*a*, *b*) correlates inversely with carotenoid accumulation in the peel and flesh; therefore, both genes are good candidates for regulating fruit color [111,157]. One of the best-characterized examples of the CCD4 role in fruit pigmentation has been described in peach (*Prunus persica*). White-fleshed peach cultivars contain at least 10 times less carotenoid concentration and higher nor-isoprenoid carotenoid-derived volatiles than the yellow ones [158]. Recently, several studies have established that alterations in the *CCD4* gene are directly related to carotenoid content in yellow peach varieties. In yellow cultivars, *CCD4* gene expression is significantly reduced, the formation of a truncated CCD4 protein avoids carotenoids degradation in fruit mesocarp, or both [159,160,161,162].

In mandarins and oranges, the specific expression of the *CCD4b1/CCD4* gene in the fruit peel during ripening is responsible for the asymmetric cleavage of β-cryptoxanthin and zeaxanthin to generate the C_30_ apocarotenoids 3-OH-β-8-apocarotenal (β-citraurin) and β-apo-8-apocarotenal [131,133], respectively. It is interesting to note that, due to the intense orange-red color of these C_30_ apocarotenoids, the citrus CCD4 activity enhances fruit pigmentation, in contrast to the other CCD4s described so far whose activity is associated with color loss. In other species, such as bitter melon, goji berry, and tomato, the expression of at least one CCD4 gene has been reported in ripened fruits, but no relationship has been established with carotenoid content [148,155,163]. In potato tubers, compared with the yellow ones, CCD4 activity regulates coloration, as indicated by the elevated *CCD4* expression in mature white-fleshed tubers [150]. Moreover, in CCD4 RNAi potato lines, total carotenoid content was enhanced in tubers (up to 5.6-fold) and flowers, but not in other plant organs [150]. The function of this enzyme in tubers is probably associated with stress response since the potato tubers from RNAi lines showed diverse developmental alterations linked to heat stress phenotypes [150]. Recently, it has been proposed that an apocarotenoid derived from β-carotene in potato tubers could act as a signaling molecule in stress responses [153].

In other plant species, CCD4 expression is also modulated by specific abiotic stresses. Saffron *CCD4a* and *b* are expressed in leaf tissues upon dehydration or heat stress as well as in senescence leaves [89]. Moreover, *CCD4c* is upregulated in flower stigmas subjected to different stress treatments, such as osmotic, wounding, and cold or heat stresses [130]. In *B. rapa* and *B. oleracea* seedlings, *CCD4* transcript levels increase in response to different abiotic stresses as well as to exogenous treatments with SLs and ABA phytohormones, suggesting their involvement in plant stress resistance response [152]. In other processes involving cellular responses to stress, such as dark-induced leaf senescence or seed maturation and desiccation, a major role of CCD4 in carotenoid degradation has been proposed [107]. These processes are associated with severe alterations in chloroplast structures, causing disassembly of photosystems and light-harvesting complexes and the release of carotenoids, in which CCD4 activity may play a crucial role in modulating turnover of β,β-carotenoids. By contrast, high-light stress in *Arabidopsis* leaves causes a strong downregulation in CCD4 expression [164], which may prevent cleavage of xanthophylls with a photoprotective function [134]. A role of CCD4 maintaining carotenoid homeostasis has also been reported in *Arabidopsis* overexpressing *PSY* [134]. In these plants, CCD4 activity is essential for the formation of apocarotenoid (C_13_) glycosides (derived from epoxidized xanthophylls) in leaves and long-chain apocarotenoids (derived from β-carotene) in roots. Moreover, a lethal phenotype was observed in *PSY*-overexpressing seedlings defective in CCD4 activity, supporting the key role of CCD4 as a first step in detoxifying excess of carotenoids into apocarotenoid glycosides [134]. The CCD4 activity is also involved in the formation of an uncharacterized apocarotenoid, most likely derived from ζ-carotene, phytofluene, or both, participating in a novel signaling process that regulates early chloroplasts and leaf development [135].

In summary, the presence of different CCD4 members with tissue- and organ-specific expression patterns in many plant species indicates a high functional diversification within this subfamily. In the last few years, we have gained much knowledge on the physiological, biochemical, and molecular regulation of CCD4 enzymes and their presumed involvements in different processes. In one case, the function is concerned with the production of apocarotenoids in flowers and fruits to attract pollinators and seed dispersers, which has eco-physiological and reproductive implications. In another case, the role is directly involved in carotenoid turnover, related to specific stress conditions and alterations of the carotenoid pathway. A third function was recently identified with the involvement of CCD4 activity in the formation of an apocarotenoid-derived signal that regulates early chloroplasts and leaf development. Future research on different CCD4 members in multigene families, including structural, functional, and spatial studies as well potential interactions with other enzymes of the carotenoid pathway, will help towards a better understanding as to how carotenoid content is modulated in different plant organs.

### 4.3. The CCD7 and CCD8 Subfamilies

The first report on CCD7 and CCD8 enzymes in plants derives from the *max4* mutant of *Arabidopsis*, affected in the AtCCD8 gene (Figure 1). The mutants exhibit an increase in lateral branching, which reminiscent of the phenotype of the *max3* mutant, which shows an increase in lateral branching as a result of a mutation in the *AtCCD7* gene, indicating that both AtCCD8 and AtCCD7 are involved in this developmental process [165,166]. The biochemical characterization of AtCCD7 and AtCCD8 [167] demonstrated that AtCCD7 catalyzes a C9–C10 cleavage of β-carotene to produce 10′-apo-β-carotenal (C_27_) and β-ionone (C_13_). The AtCCD8 protein is able to catalyze a secondary cleavage of 10′-apo-β-carotenal at the C13–C14 position to produce 13-apo-β-carotenone (C_18_). However, it has been shown that CCD7 is also active on many other carotenoid substrates [166,167]. In contrast to CCD1, which has a cytoplasmatic location, CCD7 and CCD8 are localized in plastids, as CCD4 and CCD2 are, the predominant sites for carotenoid accumulation [87].

**Figure 4:**
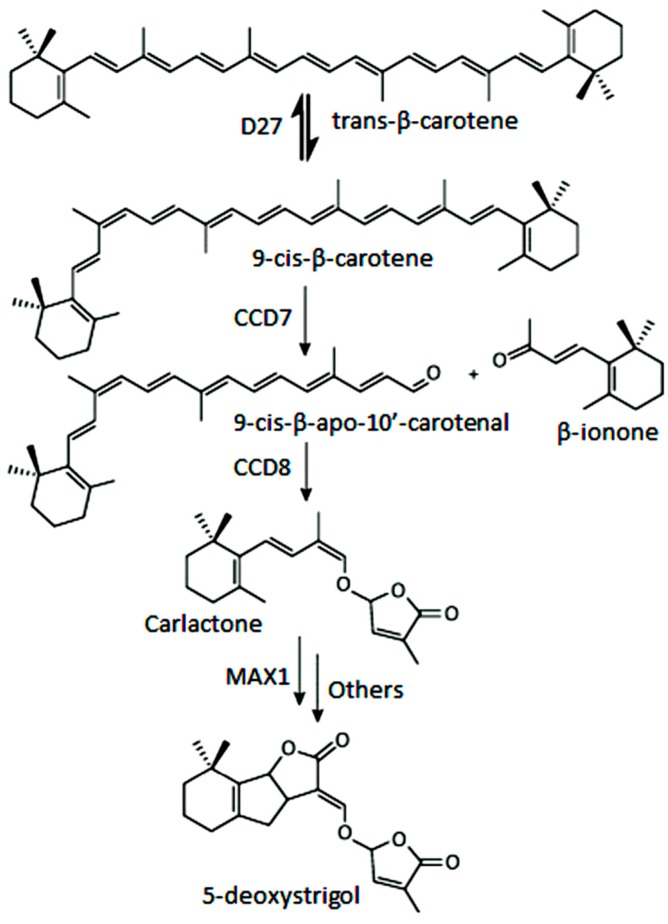
Biosynthetic pathway of strigolactones (SLs) from β-carotene.

The CCD7 and CCD8 enzymes are involved in the biosynthesis of a relatively novel class of apocarotenoid hormones, the strigolactones (SLs), which control lateral shoot growth and which appear to be well conserved among the studied plant species. Branching mutants from *Arabidopsis*, petunia, pea, and rice lacking CCD7 or CCD8 have reduced SL concentrations, and applications of synthetic SLs to the mutant restores the wild-type branching phenotype [87,168,169]. In tomato, a reduction of CCD7 expression increases branching [170]. This is the case of the tomato mutant Sl-ORT1, which is deficient in SLs and has reduced CCD7 expression [171]. These enzymes have been identified in all high plant genomes sequenced up to date. SLs are derived from carotenoids, thus belonging to the class of the apocarotenoids that function as signaling molecules. This is exemplified by their role promoting germination of the parasitic plants *Striga* and *Orobanche* [172] or their involvement in symbiotic interaction with arbuscular mycorrhizal fungi [173]. CCD7 and CCD8 are involved in sequential cleavage reactions needed for the synthesis of SLs. These metabolites share a common C_19_ structure consisting of a tricyclic lactone connected via an enol ether bridge to a second lactone (Figure 4). The first steps of SL synthesis involve isomerization and dioxygenase-mediated cleavage of a carotenoid precursor via plastidic isomerase action by D27, followed by the cleavage activity of CCD7 and CCD8. MAX1 encodes a cytP450 enzyme predicted to act downstream of CCD7 and CCD8, and is required for the synthesis of active SLs. SL signaling requires the hormone-dependent interaction of the α/β hydrolase protein DWARF 14 (D14), a likely SL receptor, with DWARF 3 (D3), an F-box component of the Skp-Cullin-F-box (SCF) E3 ubiquitin ligase complex. The third component in the signaling pathway is D53, which shares predicted features with the class I Clp ATPase proteins and can form a complex with D14 and D3. SLs induce D53 degradation by the proteasome and abrogate its activity in promoting axillary bud outgrowth [174]. Analysis of mutants, such as D27, D17/CCD7, and D10/CCD8 from rice, showed that all these genes are involved in the biosynthesis of SLs. However, mutants lacking one or more of these genes are able to produce SL metabolites, which indicate an alternative minor pathway for SL biosynthesis [175].

The first identified function of SLs in higher plants was as a stimulatory signal in seed germination of root parasitic weeds, such as the witchweed *Striga* spp. and broomrapes (*Orobanche* and *Phelipanche* spp.) [176]. The first germination stimulant identified for *Striga* was called strigol, a member of SLs isolated from cotton (*Gossypium hirsutum* L.) root exudates [177]. Many other compounds with similar structures and with germination stimulatory properties were discovered in root exudates of host plant species [178]. *Orobanche* and *Phelipanche* spp. are obligate non-photosynthetic holoparasites that depend fully on their host for nutritional needs. It is not clear how these parasites perceive SLs. Recently, it has been suggested that MAX2, AtD14, or both, which are sensitive to SLs, might be involved in this process [179]. In rice, the obstruction of the carotenoid biosynthesis in different steps of the pathway, including those leading to CCD7 substrates, led to reduced SLs production and secretion into the rhizosphere, resulting in decreased *Striga* germination and consequently lower *Striga* infection [180].

The analysis of genomes from several plant species showed a single copy for the CCD7 gene. However, in most of the analyzed genomes, CCD8 is present as several copies [144]. In some cases, as in apple (*M. domestica*) and camelina (*Camelina sativa*), CCD8 genes are organized in tandem in the same chromosome (Tables S2 and S3). CCD7 and CCD8, as other members of CCDs genes, contain introns, with the exception of the NCED related group, which is intron-less. The CCD7 genes are characterized by the presence of 4–7 introns, four of which are well conserved among different plant species. The number of introns in CCD8 sequences ranges between 2 and 6, with 5 being the most frequent number among the species investigated. The identities among the CCD7 and CCD8 paralogous genes range between 36% and 100% and between 45% and 100%, respectively. The phylogenetic analysis of CCD7 proteins shows three clusters distinguishing the Embryophyte, Grass, and Eudicot phyla. However, a similar analysis with CCD8 proteins did not so clearly match this taxonomical classification (Figure S3).

In contrast to the high functional conservation of the CCD7 and CCD8 enzymes, their genes present different expression patterns. Previous works in some plants, e.g., in rice, indicated that CCD7 and CCD8 genes are found to be upregulated in root [181]. In the columella cap of primary and lateral roots, CCD8 is highly expressed [182,183], and exogenous treatment with auxin 1-naphthaleneacetic acid (NAA) led to an overexpression of CCD8 expression in the primary root and cortical tissue. The expression of CCD8 in roots tissues from *Arabidopsis* [87], petunia [184], pea [185], kiwi [186], and tomato [187] exceeds shoot expression. However, in saffron and chrysanthemum, the opposite behavior was detected [188]; in rose, no transcripts are found in roots [189]. High expression of CCD7 in root from *Arabidopsis* is observed, [166], but more recently even higher transcripts were observed in seeds and in the stem vascular tissue [190]. Different patterns of CCD7 expression was found in rice [191], petunia [192], and tomato [170]. High expression was detected in vascular bundle tissue in rice, while CCD7 was mainly expressed in internodes and nodes in petunia, and CCD7 expression was low in green tissue in tomato in comparison to root and shoots. These differences suggest different mechanisms of the SL-regulation of the shoot branching of the root and shoot in different species.

In addition to regulating shoot architecture and branching, SLs are involved in many other processes, including root growth, root hair elongation, lateral root formation, adventitious rooting, stem elongation, leaf expansion, leaf senescence, secondary growth, and drought and salinity responses [183,193,194,195,196]. CCD7 and CCD8 from soybean are involved in abiotic stress physiology, and their expression levels were greatly influenced by exogenous ABA treatment [112]. CCD7 from *Lotus japonicus* affects reproduction by reducing the number of flowers, fruits, and seeds and modulates leaf senescence and abscission [197]. CCD8 from kiwifruit modifies branch development and slows up leaf senescence [186]. An analogous association was detected in the rice tillering *dwarf* mutant *D3*, and the mutant *MAX2*/*ORE9* from *Arabidopsis* [198]. Nevertheless, the delayed leaf senescence was not observed in the *rms4* mutant from pea [199], suggesting that the connection between leaf senescence and branch development has followed a convergent evolution in plant species.

Recent experimental evidence has revealed that auxin influences pea mycorrhizal symbiosis by regulating the production of SL plant hormones. During the symbiosis between the auxin deficient *bsh* mutant and the mycorrhizal fungus *Glomus intraradices*, the low auxin production correlated with reduced mycorrhizal colonization, SL levels, and biosynthetic gene expression, including CCD7 and CCD8 from pea [200]. In *Crocus sativus*, CCD7 and CCD8 were identified in saffron corms and stigmas, leading to attribute novel roles to SLs in this plant [201]. In corms, SLs act synergically with auxin to arrest the outgrowth of the axillary buds. In stigma tissue, transcripts were detected at a higher level than in vascular tissues, leaves, and roots. The abundance of both transcripts in immature orange stigmas suggests that these enzymes and SLs might regulate procambial activity and the development of new vascular tissues connecting leaves with the mother corm. A similar function was already suggested for SLs in flower development in petunia [184].

There has been great progress over the past years in characterizing plant CCD enzymes and their apocarotenoid products. As more functions are unraveled for apocarotenoids in plants, more additional roles will be assigned to CCD7 and CCD8. Further characterization of these genes may provide hints about the origin of parasitism, as well as new approaches for controlling parasitic plants and understanding important physiological processes of the underlying metabolic pathways.

## 5. CCDs in Algae

Algae are classified throughout many divisions of the kingdom Plantae. Their sizes range from single cells of picophytoplankton to seaweeds. Algae can synthesize many kinds of carotenoids that are absent in higher plants and therefore have been proposed as excellent chemotaxonomic markers [202,203]. Only few studies have been conducted with CCDs in algae. The best-known apocarotenoid in algae is retinal [204,205]. Most unicellular flagellate algae are phototactic [206]. They have developed an eyespot, which have been most studied in *Chlamydomonas reinhardtii*. The eye of Chlamydomonas comprises the optical system and at least five different rhodopsin photoreceptors. Two of these receptors are rhodopsin-ion channel hybrids switching between closed and open states by photoisomerization of an attached retinal chromophore [207]. The proteome of the eyespot apparatus in *C. reinhardtii* includes putative carotenoid cleavage dioxygenases homologous to *Synechocystis* sp. PCC 6803 ACOX_SYNY3 [208], which forms retinal from diverse apo-carotenoids in vitro [29]. However, the phylogenetic analysis of the CCD sequences present in the databases from algae did not show the presence of such homologues; instead, only CCD7 and CCD8 homologues have been identified (Table S2, Figure S4). As mentioned in the former section, SLs comprise an important class of apocarotenoid derivatives acting as hormones in plants [209] and have also been found in several algae [210]. A recent study showed that freshwater green algae belonging to the Charales, some of the closest freshwater green algal relatives of land plants, produce and exude SLs. The same study showed the presence of CCD7 and CCD8 homologues in different green algae taxa (Table S2, Figure S4) [211]. The authors suggested that SLs could play a role in rhizoid elongation in algae and thus increase their anchorage ability.

Other important apocarotenoids in algae are the carotenoid-derived volatiles, which are released by diverse algae taxa and influence aquatic odors [212]. *Ulva prolifera*, *Ulva linza*, *Monostroma nitidum*, *Ulothrix fimbriata*, and *Porphyra tenera* produce volatile apocarotenoids in a high proportion. These C_13_ apocarotenoids or their derivatives exhibit growth-regulating properties in algae [213]. In addition, they may play ecological roles in providing competitive advantages, e.g., by inhibiting the growth of surrounding phytoplankton [214]. Functional carotenoid cleavage-like enzymes are expected to contribute to the formation of volatile apocarotenoids in these macro-algae [215]. No specific CCD has been isolated and identified as responsible for the production of these apocarotenoids, but the presence of homologous sequences closely related to CCD1 and CCD4 subfamilies (Table S2, Figure S4) suggests that theses CCDs could be good candidates to mediate the formation of these apocarotenoids volatiles.

## 6. Conclusions

In nearly 20 years, the research on CCDs enzymes has evolved quickly, with the cloning of many different CCDs from plants and microorganisms. The increasing number of biochemical analyses for newly identified CCDs evidences the functional diversification of these enzymes in different species, adapted to a diversity of substrates and cleaving sites in a large variety of organisms. More recently, the knowledge on new putative CCDs is increasing enormously with the breakthrough of genome sequencing projects and transcriptome analyses of many additional species. The experimental basis for their future characterization has been solidly established, and the functional diversity found so far allows anticipating that new carotenoid or apocarotenoid substrates for novel CCD enzymatic reactions will be discovered in the next years. The biological purposes of such reactions will be predictably more elusive. Although the function of several novel CCDs has been resolved by in vitro analysis, we are far from understanding the activities and function of these enzymes in vivo in the different organisms, and more efforts will be needed to learn about how these CCDs are regulated at metabolic, transcriptional, and translational levels. The functions for newly identified CCDs and apocarotenoid products will be a future challenge, and effective research will require integrated multidisciplinary approaches to advance in this fascinating field of research.

## Figures and Tables

**Table 1:** Enzymatic activity of bacterial apo-carotenoid cleavage oxygenases Diox1 and NosAco showing products to different substrates.

Substrates	Diox1 *Synechocystis* sp. PCC 6803	NosAco *Nostoc* sp. PCC 7120
β-apo-4′-carotenal 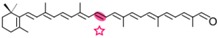	Retinal	Not detected
β-apo-8′-carotenal 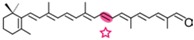	Retinal	Retinal
β-apo-10′-carotenal 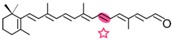	Retinal	Retinal
β-apo-12′-carotenal 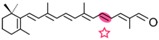	Retinal	Not detected
(3*R*)-3-OH-β-apo-8′-carotenal 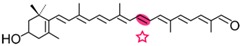	3-OH-retinal	3R-3-OH-retinal
(3*R*)-3-OH-β-apo-12′-carotenal 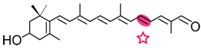	3-OH-retinal	3R-3-OH-retinal
Apo-8′-lycopenal 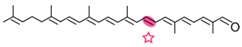	Acycloretinal (slow)	Acycloretinal
Apo-10′-lycopenal 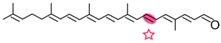	No information	Acycloretinal
β-apo-8′-carotenol 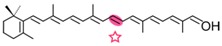	Retinal	Retinal
(3*R*)-3-OH-β-apo-8′-carotenol 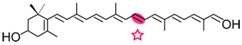	3-OH-retinal	3-OH-retinal
Apo-8′-lycopenol 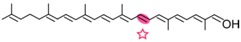	No information	Acycloretinal
4-oxo-β-apo-8′-carotenal 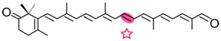	4-oxo-retinal (slow)	Not tested
References	[29]	[32]

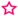
: Enzyme cleaves at the C15–C15′ double bond, or equivalent positions.

**Table 2:** Enzimatic activity and substrate specificity of different bacterial carotenoid oxygenases.

Assayed Substrates	Enzymes and Products						
	β-carotene-oxygenase *Microcystis* PCC 7806	NosCCD (NSC1) *Nostoc* sp. PCC 7120	MtCCO *Mycobacterium tuberculosis*	NSC3 *Nostoc* sp. PCC 7120	NACOX1 *Novosphingobium*	SaCCO *Sphingopyxis alaskensis*	PpCCO *Plesiocystis pacifica*
β-apo-8′-carotenal 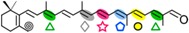		β-ionone ( 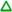 ) + Apo-8,10′-apocarotene-dial ( 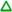 ) Other minority products ( 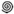 , 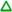 )	**β-apo-13-carotenone** ( 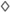 ) Retinal ( 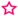 )	β-apo-13-carotenone ( 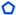 ) β-apo-14′-carotenal Transretinal ( 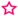 )	β-apo-13-carotenone ( 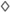 )	Apo-12′-carotenal ( 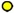 ) Apo-10′-carotenal ( 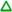 )	
β-apo-10′-carotenal 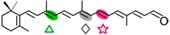		β-ionone ( 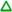 ) + Apo-10,10′-apocarotene-dial ( 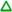 )	β**-apo-13-carotenone** ( 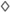 ) Retinal ( 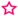 )				
3-OH-β-apo-8′-carotenal 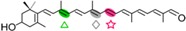		3-OH-β-ionone ( 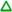 ) + Apo-8,10′-apocarotene-dial ( 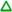 )	3-OH-β-apo-13-carotenone ( 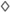 ) **3-OH-retinal** ( 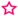 )				
3-OH-β-apo-10′-carotenal 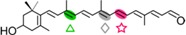		3-OH-β-ionone ( 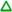 ) + Apo-10,10′-apocarotene-dial ( 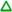 )	3-OH-β-apo-13-carotenone ( 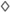 ) **3-OH-retinal** ( 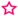 )				
γ-carotene 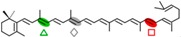		β-ionone ( 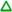 ) + Apo-8,10′-apocarotene-dial ( 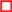 )			β-apo-13-carotenone ( 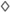 )		
β-carotene 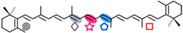	2 × β-cyclocitral ( 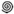 , 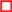 ) Crocetindial ( 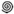 , 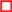 )	Apo-10,10′-apocarotene-dial ( 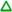 )	β-apo-13-carotenone ( 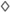 ) Retinal ( 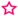 ) β-apo-14′-carotenal ( 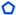 )	No	No	No (in vitro)	No
Zeaxanthin 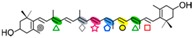	2 × hydroxi-β-cyclocitral ( 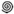 , 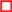 ) Crocetindial ( 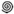 , 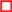 )	3-OH-β-ionone ( 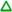 ) Apo-10,10′-apocarotene-dial ( 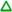 )	3-OH-β-apo-13-carotenone ( 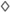 ) 3-OH-β-apo-14′-carotenal ( 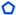 ) 3-OH-retinal ( 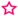 ) 3-OH-β-apo-11-carotenal?	No	No	No (in vitro)	Apo-13′-zeaxanthinone ( 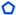 ) Apo-14′-zeaxanthinal ( 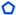 ) Apo-12′-zeaxanthinal ( 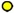 )
Lycopene 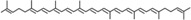				No	No	poorly	Yes (in vivo) Unknown product
Lutein 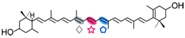			3-OH-β-apo-13-carotenone ( 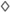 ) 3-OH-β-apo-14′-carotenal ( 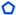 ) 3-OH-retinal ( 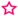 ) 3-OH-α-apo-15′-carotenal( 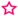 )		No		
Echinenone 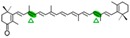	No	4-oxo-β-ionone ( 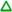 ) Apo-10,10′-apocarotene-dial ( 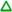 ) β-ionone ( 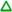 )					
Canthaxantin 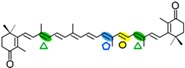		2 x 4-oxo-β-ionone ( 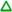 ) Apo-10,10′-apocarotene-dial ( 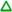 )					Apo-13′-canta-xanthinone ( 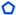 ) Apo-14′-cantaxanthinal ( 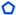 ) Apo-12′-cantaxanthinal ( 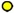 )
Astaxanthin 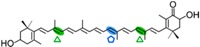		3-OH-β-ionone ( 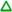 ) 3-OH, 4-oxo-β-inone ( 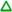 ) Apo-10,10′-apocarotene-dial ( 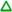 )					Apo-13′-astaxanthinone ( 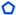 ) Apo-14′-astaxanthinal ( 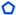 )
References	[19]	[30,33]	[36]	[31]	[37]	[38]	[38]

Symbols indicate double bonds where oxygenases cleave. 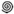: C7–C8; 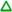: C9–C10; 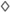: C13–C14; 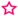: C15–C15′; 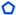: C13′–C14′; 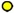: C11′-C12′; 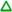: C9′–C10′; 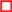: C7′–C8′. When an enzyme cleaves at different positions, the predominant product is indicated in bold. Lack of bold means no product preference or information not available.

**Table 3:** Substrates tested and cleaved with a unique oxygenase.

Substrates	Oxygenase	Products
β-apo-4′-carotenal 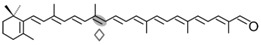	NACOX1	β-apo-13-carotenone ( 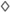 )
3,3′-dihydroxy-isorenieratene 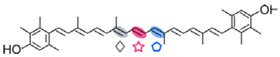	MtCCO	3-OH-β-apo-13-carotenone ( 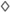 ) 3-OH-β-apo-15′-carotenal ( 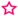 ) 3-OH-β-apo-14′-carotenal ( 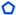 )
Echinenone 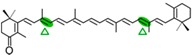	NosCCD	Apo-10,10′-apocarotenoid ( 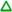 ) 2 × C_13_ ( 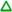 )
Nostoxanthin 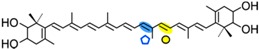	PpCCO	Apo-14′-nostoxanthinal ( 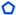 ) Apo-12′-nostoxanthinal ( 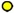 )
Hydroxylycopene 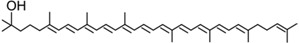	PpCCO	Unknown
Dihydroxylycopene 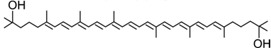	PpCCO	Unknown
Torulene 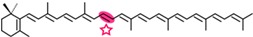	NSC3	Transretinal ( 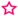 )
4,4′-diapotorulene 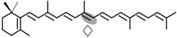	NSC3	Apo-14′-diapotorulenal ( 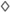 )
4,4′-diapotorulene-4-al 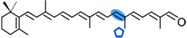	NSC3	Apo-10′-diapotorulenal ( 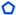 )
4,4′-diaponeurosporene 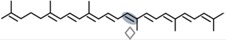	NSC3	Apo-14′-diaponeurosporenal ( 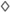 )
4,4′-diaponeurosporen-4′-al 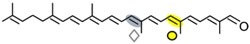	NSC3	Apo-14′-diaponeurosporenal ( 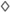 ) Apo-10′-diaponeurosporenal ( 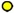 )
4,4′-diaponeurosporen-4′-oic acid 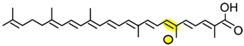	NSC3	Apo-10′-diaponeurosporenal ( 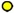 )
Myxol 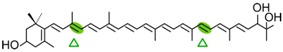	NosCCD	3-OH-β-ionone ( 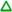 ) Apo-8′-10-apocarotenedial ( 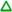 )
Myxol-2′-fucoside 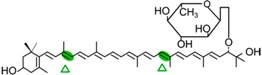	NosCCD	3-OH-β-ionone ( 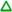 ) Apo-8′-10-apocarotenedial ( 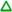 ) acetate adduct C10
4-ketomyxol-2′-fucoside 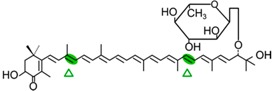	NosCCD	3-OH-4-oxo-β-ionone ( 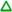 ) Apo-8′-10-apocarotenedial ( 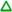 )
4-hydroxymyxol-2′-fucoside 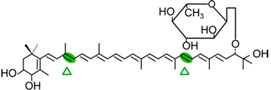	NosCCD	3, 4-OH-β-ionone ( 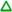 ) Apo-8′-10-apocarotenedial ( 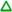 )

Symbols indicate double bonds where oxygenases cleave. 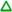: C9=C10; 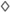: C13=C14; 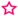: C15=C15′; 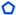: C13′=C14′; 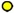: C11′=C12′; 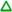: C9′=C10′; 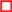: C7′=C8′.

**Table 4:** Functional characterization of CCD4 enzymes.

Species	Protein	Assay ^a^	Parent Carotenoid	Cleavage Position	Product Detected	References
*Arabidopsis thaliana*	CCD4	in planta	β-carotene, Violaxanthin	n.d.	n.d.	[107]
		in planta	Phytofluene, ζ-carotene	n.d.	n.d.	[135]
		in planta (leaf)	Epoxy-β-xanthophylls	C9–C10 or C9′–C10′	C_13_-glycosids	[134]
		in planta (root)	β-carotene	n.d.	long-chain free apocarotenals (C_15_ to C_30_)	[134]
		in vitro	Apo-β-caroten-8′-al	C9–C10	β-ionone	[96]
*Brassica napus*	CCD4_C3	in planta	α-carotene, δ-carotene	C9–C10	α-ionone	[152]
*Chrysantemum morifolium*	CCD4a	in vivo and in vitro	β-carotene	C9–C10 (C9′–C10′)	β-ionone	[96]
*Citrus clementina*	CCD4b1	in vitro	β-carotene, β-cryptoxanthin, Zeaxanthin, Lutein, α-carotene	C7–C8 or C7′–C8′	3-OH-apo-β-8-carotenal, apo-β-8-carotenal (C_30_); β-cyclocitral and 3-OH-β-cyclocitral (C_10_)	[133]
*Citrus unshiu*	CCD4	in vitro and in vivo	β-cryptoxanthin, Zeaxanthin	C7–C8 or C7′–C8′	3-OH-apo-β-8-carotenal	[131]
*Crocus sativus*	CCD4a/b	in vivo	β-carotene, Zeaxanthin	C9–C10 (C9′–C10′)	β-ionone, β-OH-ionone	[89,147]
	CCD4c	in vivo	β-carotene	C9–C10 or C9′–C10′	β-ionone, β-cyclocitral	[130]
		in planta		C7–C8 or C7′–C8′		
		in planta	Lutein	C9–C10	Megastigma-4,6,8-triene (derived from 3-OH-α-ionone)	[130]
*Malus domestica*	CCD4	in vivo	β-carotene	C9–C10 (C9′–C10′)	β-ionone	[96]
*Osmanthus fragans*	CCD4	in vivo	β-carotene	C9–C10 (C9′–C10′)	β-ionone	[96]
*Rosa damascena*	CCD4	in vivo	β-carotene	C9–C10 (C9′–C10′)	β-ionone	[96]
		in vitro	apo-β-8-carotenal	C9–C10	β-ionone	[96]
*Solanum tuberosum*	CCD4	in planta	Violaxanthin	n.d.	n.d.	[150]
		in vitro and in vivo	β-carotene	C9–C10 or C9′–C10′	Apo-β-caroten-10′-al; β-ionone	[153]
		in vitro	α-carotene, Lutein, Zeaxanthin, β-cryptoxanthin	C9–C10 or C9′–C10′	3-OH-β-apo-10′-carotenal, β-apo-10′-carotenal3-OH-ε-apo-10´-carotenal	[153]
*Vitis vinifera*	CCD4a/b	in vivo	ε-carotene,	C9–C10 (C9′–C10′)	α-ionone,	[98]
	CCD4a/b	in vivo	Neurosporene	C9′–C10′	Geranylacetone	[98]
	CCD4a/b	in vivo	Lycopene	C5–C6 (C5′–C6′)	6-methyl-5-hepten-2-one	[98]
	CCD4b	in vivo	ζ-carotene	C9–C10 (C9′–C10′)	α-ionone, Geranylacetone	[98]

^a^ In planta refers to data inferred from changes in carotenoid and/or apocarotenoid profiles in overexpressing or knockout CCD4 mutants; In vivo assay indicates data obtained from bacteria over-accumulating carotenoids and expressing CCD4; in vitro assay refers to enzymatic assays performed with recombinant CCD4 enzyme. n.d. not determined.

## Supplementary Materials

Supplementary materials can be found at www.mdpi.com/1422-0067/17/11/1781/s1.
