# Supplementary material for: Carotenoid Cleavage Oxygenases from Microbes and Photosynthetic Organisms: Features and Functions
Source: Int J Mol Sci. 2016 Oct 26;17(11):1781. doi: 10.3390/ijms17111781 (PMC5133782; doi:10.3390/ijms17111781)
Supplement: Supplementary file 1 [file ijms-17-01781-s001.pdf]

# Supplementary Materials: Carotenoid Cleavage Oxygenases from Microbes and Photosynthetic Organisms: Features and Functions

**Oussama Ahrazem, Lourdes Gómez-Gómez, María J. Rodrigo, Javier Avalos and María Carmen Limón**

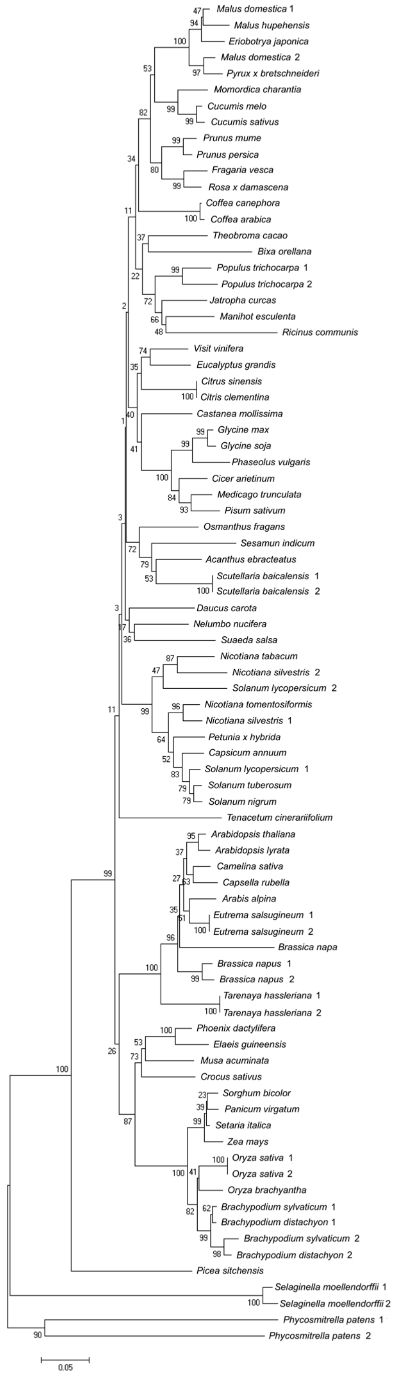


**Figure S1.** Phylogenetic tree of CCD1 enzymes. The analysed proteins are those presented in Table S1. Proteins were aligned using ClustalW and tree was generated using the Neighbor-Joining method. The optimal tree is represented and the percentages of replicated trees in which the associated proteins clustered together in the bootstrap test (2500 replicates) are shown next to the branches. The tree is drawn to scale with branch lengths in the same units as those of the evolutionary distances used to infer the phylogenetic tree. Analysis was conducted in MEGA6.

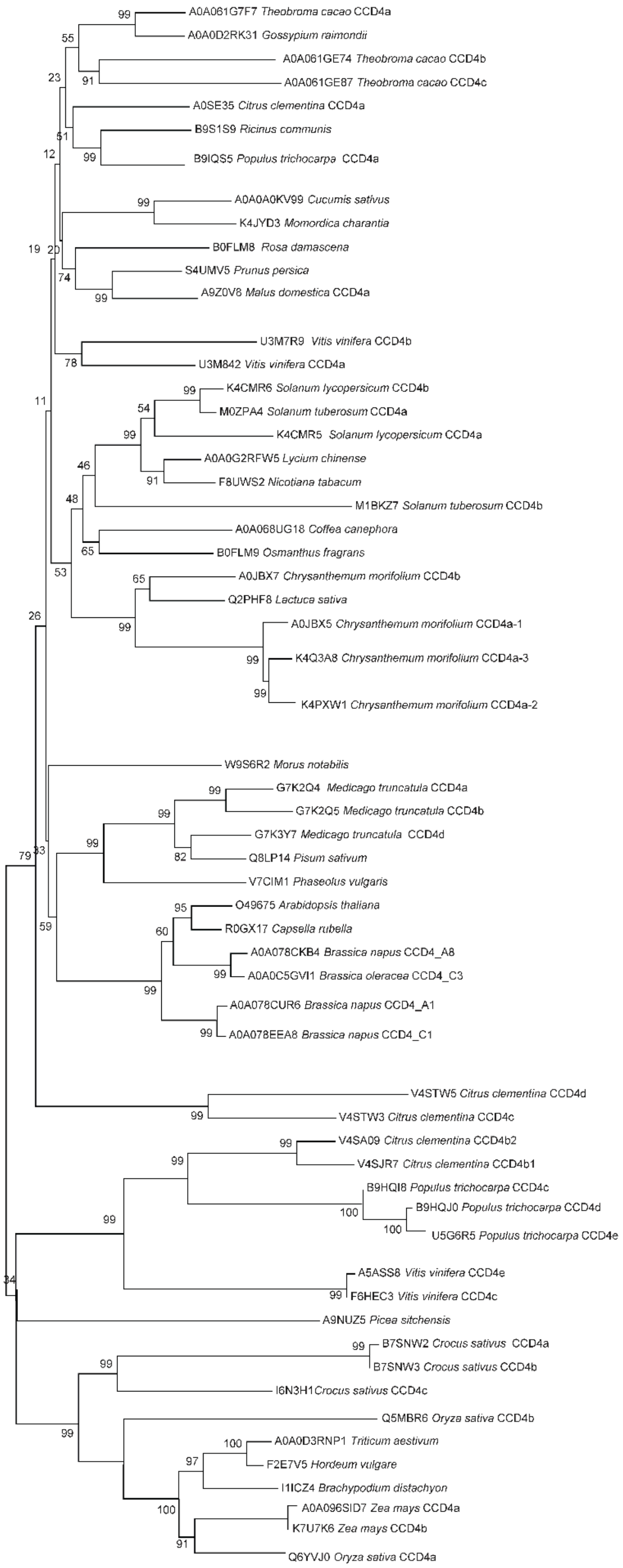


**Figure S2.** Phylogenetic tree of CCD4 enzymes. A total of 59 proteins were selected from the UniProtKB based on sequence similarity to *Arabidopsis* CCD4 and used for the analysis. Methods for proteins alignment and tree generation are described in the legend of Figure S1. UniProtKB accession numbers are indicated.

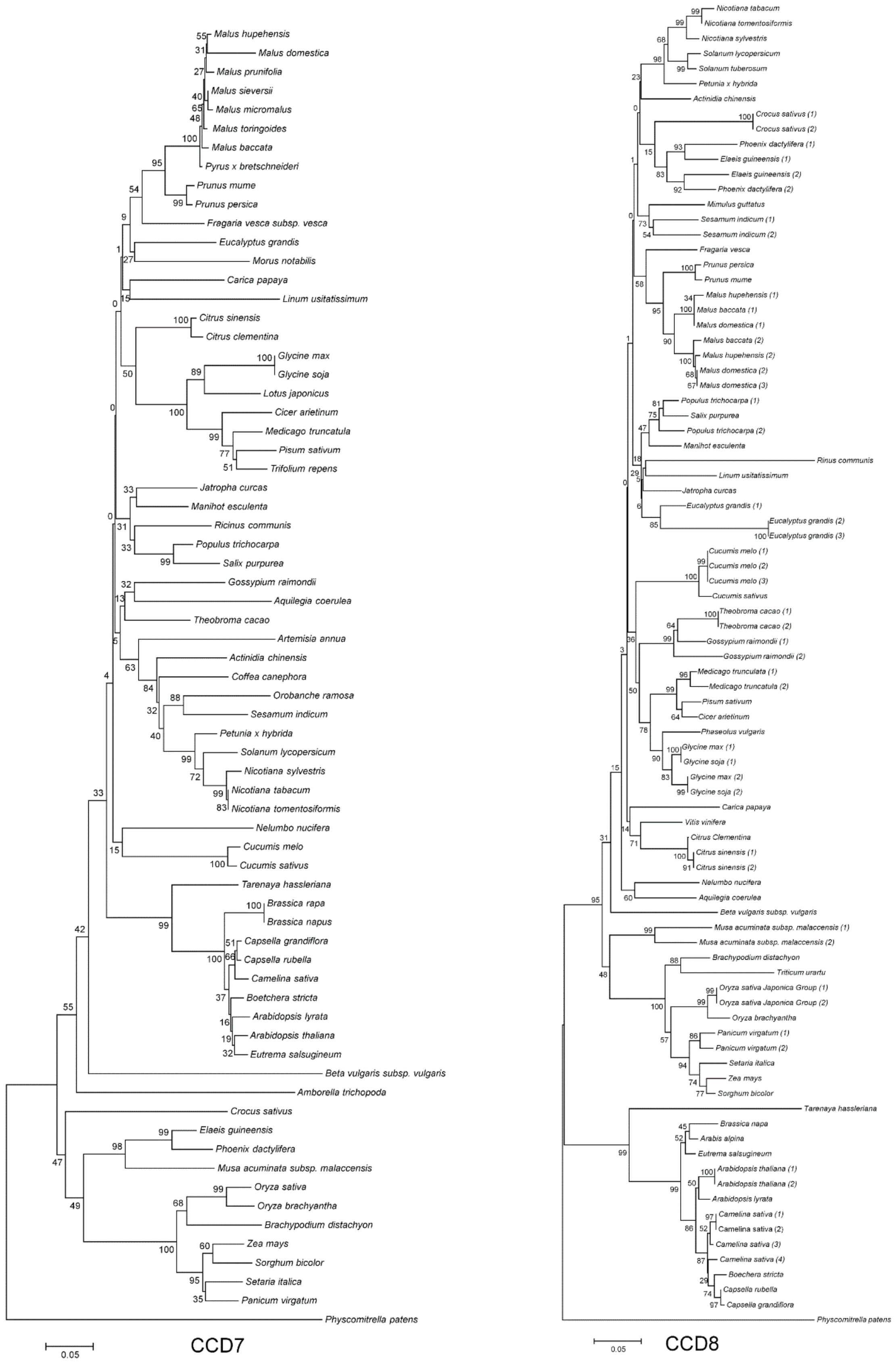


**Figure S3.** Phylogenetic tree of CCD7 and CCD8 enzymes. The analysed proteins are those presented in Tables S2 and S3. Methods for proteins alignment and tree generation are described in the legend of Figure S1.

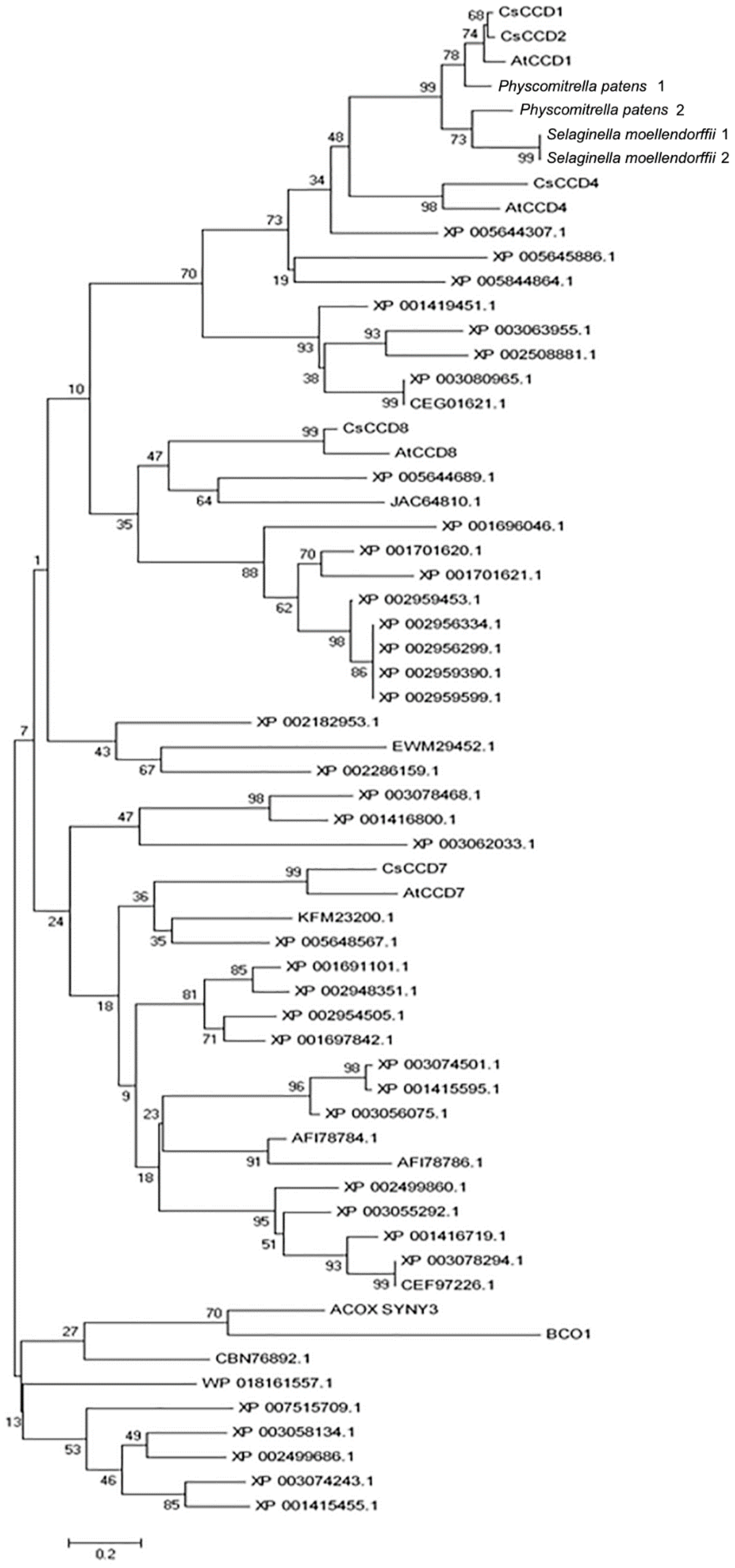


**Figure S4.** Phylogenetic tree of algal CCD enzymes. Methods for proteins alignment and tree generation are described in the legend of Figure S1.

**Table S1.** CCD1 genes and proteins present in different plant species.

| Species | Protein Code | Intron Number | Gene Bank | Chromosome Number |
|---|---|---|---|---|
| *Acanthus ebracteatus* | AFC88468. | | | |
| *Arabidopsis lyrata* | XP_002876727.1 | 14 | NW_003302551.1 | 3 |
| *Arabidopsis thaliana* | NP_191911.1 | 13 | AT3G63520.1 | 3 |
| *Arabis alpina* | KFK35504.1 | 13 | CM002873.1 | |
| *Beta vulgaris* | XP_010695196.1 | 13 | NC_025813.1 | 2 |
| *Brachypodium distachyon* | XP_003577313.1 | 13 | NC_016134.1 | 4 tandem |
| | XP_003577349.1 | 13 | NC_016134.1 | 4 |
| *Brachypodium sylvaticum* | ACO87672.1 | 13 | FJ234838.1 | tandem |
| | ACO87673.1 | 13 | FJ234838.1 | |
| *Brassica rapa* | XP_009117091 | 13 | NC_024803 | A9 |
| ***Camelia sativa*** | XP_010512783.1 | 13 | NC_026891 | 5 |
| | XP_010413131.1 | 19 | NC_025691.1 | 7 |
| | XP_010413132.1 | | | |
| *Capsicum annuum* | CAC79644.1 | | | |
| *Castanea mollisima* | ABO69703.1 | | | |
| ***Cicer arietinum*** | XP_004510991.1 | 13 | NC_021166.1 | Ca7 |
| | XP_004510990.1 | | | |
| ***Citrus clementina*** | XP_006439107.1 | 14 | NW_006262274.1 | |
| | XP_006439108.1 | | | |
| ***Citrus sinensis*** | XP_006482868.1 | 13 | NC_023052.1 | 7 |
| | XP_006482869.1 | | | |
| | XP_006482870.1 | | | |
| *Coffea arabica* | ABA43904.1 | | | |
| *Coffea canephora* | ABA43900.1 | 13 | GSCOC_T00025171001 | 2 |
| *Crocus sativus* | Q84KG5.1 | | | |
| *Cryptomeria japonica* | BAF31905 | | | |
| ***Cucumis melo*** | XP_008461300.1 | 13 | NW_007546327.1 | |
| *Cucumis sativus* | | | | |
| *Daucus carota* | ABB52081.1 | | | |
| *Elaeis guineensis* | XP_010931015.1 | 13 | NC_026001 | 9 |
| *Eriobotrya japonica* | AFG30025.1 | | | |
| *Eualiptus grandis* | XP_010067867.1 | 13 | GO3374 | tandem |
| | KCW66101.1 | 13 | GO3362 | |
| ***Eutrema salsugineum*** | XP_006402261.1 | 13 | NW_006256838.1 | |
| | XP_006402262.1 | | | |
| | XP_006402263.1 | | | |
| | XP_006402264.1 | | | |
| ***Fragaria vesca*** | XP_004306539.1 | 13 | NC_020497.1 | LG7 |
| *Glycine max* | XP_003542847.1 | 13 | NC_0161001 | 13 |
| *Glycine soja* | KHN19457 | 13 | KN659065.1 | |
| *Jatropha curcas* | KDP43480.1 | 13 | KK914267.1 | |
| *Lactuca sativa* | BAE72095.1 | | | |
| *Malus domestica* | XP_008375177.1 | 13 | NC_024245.1 | 7 |
| | XP_008363317.1 | 13 | NW_007545925.1 | 2 |
| | XP_008349836.1 | 9 | NC_024240 | 2 |
| *Malus hupehensis* | ACF75911.1 | | | |
| *Manihot sculenta* | ADN65332.1 | | | |
| *Medicago truncatula* | CAR57918.1 | | | |
| *Momordica charantia* | AFU91489.1 | | | |
| *Morus notabilis* | XP_010088226.1 | 13 | NW_010357396 | |
| *Musa acuminata* | XP_009394395.1 | 14 | NC_025204.1 | 3 |
| ***Nelumbo nucifera*** | XP_010241260.1 | 14 | NW_010729221.1 | |
| | XP_010241261.1 | | | |
| ***Nicotiana sylvestris*** | XP_009760607.1 | 13 | NW_009359434.1 | |
| | XP_009784260 | 13 | NW_009344641.1 | |
| | XP_009784266 | | | |
| *Nicotiana tabacum* | AIL30506.1 | | | |

**Table S1.** *Cont.*

| Species | Protein Code | Intron Number | Gene Bank | Chromosome Number |
|---|---|---|---|---|
| ***Nicotiana tomentosiformis*** | XP_009589876.1 | 13 | NW_008927330.1 | |
| | XP_009589877.1 | | | |
| | XP_009589878.1 | | | |
| | XP_009589879.1 | | | |
| | XP_009589880.1 | | | |
| | XP_009589881.1 | | | |
| *Oryza sativa* | ABA99624 | 13 | | |
| | ABA99623 | 13 | | |
| *Oryza branchiata* | XP_006661114.1 | 12 | NC_023171.1 | 9 |
| *Osmanthus fragans* | BAJ05401.1 | | | |
| *Panicum virgatum* | Pavirv00030688m | 12 | Pavirv00030688m | |
| *Petunia x hybrida* | AAT68189.1 | | | |
| *Phaseolus vulgaris* | Q94IR2 | | | |
| *Phoenix dactylifera* | XP_008801952.1 | 13 | NW_008246719.1 | |
| *Physcomitrella patents* | XP_001771028.1 | 12 | Pp1s132_26V6 | |
| | XP_001754114.1 | 14 | Pp1s12_317V6 | |
| *Picea glauca* | BT115844.1 | | | |
| *Picea sitchensis* | ABK24523.1 | | | |
| *Pirux x bretschneideri* | XP_009365069.1 | 12 | NW_008988158.1 | |
| | XP_009367500.1 | 12 | NW_008988186.1 | |
| *Pisum sativum* | Q8LP17 | | | |
| ***Populus trichocarpa*** | XP_006379094.1 | 13 | NC_008475.2 | LGIX |
| | XP_002298429.2 | 13 | NC_008467.2 | LGI tandem |
| | XP_002298430.2 | | | |
| ***Populus euphratica*** | XP_011019809 | 12 | NW_011499853.1 | |
| | XP_011012510.1 | 12 | NW_011500894.1 | |
| | XP_011005228.1 | 12 | NW_011500182.1 | |
| | XP_011019620.1 | 12 | NW_011499853.1 | |
| *Prunus mume* | XP_008231253.1 | 13 | NC_024130.1 | LG5 |
| *Prunus persica* | XP_007220659.1 | 13 | NW_006760324.1 | |
| *Ricinus communis* | XP_002517824.1 | 11 | NW_002994325.1 | |
| *Rosa damascena* | ABY47994.1 | | | |
| *Saccharum hybrid cultivar R570* | AGT17077.1 | 12 | SHCRBa_185_C01_R_60 | |
| *Scutellaria baicalensis* | AGN03859.1 | | | |
| *Selaginella moellendorffii* | XP_002962877.1 | 13 | 165469 | |
| | XP_002989454.1 | | | |
| *Setaria italica* | XP_004963348.1 | 13 | NW_004675963.1 | |
| ***Solanum lycopersicum*** | XP_010324092 | 13 | NC_015438.2 | 1 |
| | NP_001234542 | | | |
| *Solanum nigrum* | AHA43413.1 | | | |
| ***Solanum tuberosum*** | XP_006345441.1 | 14 | NW_006238994.1 | |
| | XP_006345442.1 | | | |
| *Sorghum bicolor* | XP_002465882.1 | 12 | Sb01g047540.1 | 1 |
| *Suaeda salsa* | AAY21819.1 | | | |
| *Tanacetum cinerariifolium* | AFV15390.1 | | | |
| ***Tarenaya hassleriana*** | XP_010552739.1 | 15 | NW_010966822.1 | |
| | XP_010552740.1 | | | |
| | XP_010552741.1 | | | |
| ***Theobroma cacao*** | XP_007052518.1 | 13 | NC_023625.1 | 1 |
| | XP_007052519.1 | | | |
| *Vitis vinifera* | AFJ94680 | 12 | JQ712833 | 13 |
| *Zea mays* | | 13 | GRMZM2G057243_T01 | 9 |

In bold, presence of spliced variants.

**Table S2.** CCD7 genes and proteins present in different plant species.

| Species | Protein Code | Intron Number | Gene Bank | Chromosome Number |
|---|---|---|---|---|
| *Actinidia chinensis* | ADP37985.1 | | ADP37985.1 | |
| *Amborella trichopoda* | XP_011621212.1 | | XM_011622910.1 | |
| *Aquilegia coerulea* | Aquca_037_00073.1<br>(Phytozome code) | 6 | | |
| *Arabidopsis lyrata* | XP_002880149.1 | 5 | XM_002880103.1 | |
| *Arabidopsis thaliana* | NP_182026.4 | 6 | NC_003071.7 | 2 |
| *Artemisia annua* | ADB64459.1 | | GQ996728.1 | |
| *Beta vulgaris* | XP_010692115.1 | | XM_010693813.1 | |
| *Boechera stricta* | Bostr.25993s0273.1<br>(Phytozome code) | 5 | | |
| ***Brachypodium distachyon*** | XP_003581501.1 | 7 | XM_003581453.2 | 5 |
| *Brassica napus* | CDY37022.1 | | LK032390.1 | |
| *Brassica rapa* | XP_009142313.1 | 6 | XM_009144065.1 | |
| *Camelina sativa* | XP_010518109.1 | | XM_010519807.1 | |
| *Capsella grandiflora* | Cagra.0239s0052.1<br>(Phytozome code) | 5 | | |
| *Capsella rubella* | XP_006295645.1 | 5 | XM_006295583.1 | |
| *Carica papaya* | evm.model.supercontig_22.7<br>(Phytozome code) | 6 | | |
| *Cicer arietinum* | XP_004513935.1 | | XM_004513878.1 | |
| *Citrus clementina* | XP_006425527.1 | 6 | XM_006425464.1 | |
| *Citrus sinensis* | XP_006467157.1 | 6 | XM_006467094.1 | |
| *Coffea canephora* | CDP09468.1 | | HG739122.1 | |
| *Crocus sativus* | AIF27228.1 | | KJ361477.1 | |
| *Cucumis melo* | NP_001284427.1 | | NM_001297498.1 | |
| *Cucumis sativus* | XP_004140624.1 | | XM_004140576.2 | 6 |
| *Elaeis guineensis* | XP_010934132.1 | | XM_010935830.1 | |
| *Eucalyptus grandis* | KCW79101.1 | 6 | KK198755.1 | |
| *Eucalyptus grandis* | XP_010056416.1 | | XM_010058114.1 | |
| *Eutrema salsugineum* | XP_006397680.1 | 6 | XM_006397617.1 | |
| *Fragaria vesca* | XP_004306976.2 | 4 | XM_004306928.2 | |
| *Glycine max* | ADK26570.1 | 6 | HM366150.1 | |
| *Glycine soja* | KHN22759.1 | | KN656920.1 | |
| *Gossypium raimondii* | KJB49230.1 | 5 | CM001747.1 | 8 |
| *Jatropha curcas* | XP_012083689.1 | | XM_012228299.1 | |
| *Linum usitatissimum* | Lus10021241<br>(Phytozome code) | 5 | | |
| *Lotus japonicus* | ADM88552.1 | | GU441766.1 | |
| *Malus baccata* | AHJ78579.1 | | KF887978.1 | |
| *Malus domestica* | XP_008363204.1 | 7 | XM_008364982.1 | 2 |
| *Malus hupehensis* | AHJ78580.1 | | KF887979.1 | |
| *Malus micromalus* | AHJ78576.1 | | KF887975.1 | |
| *Malus prunifolia* | AHJ78582.1 | | KF887981.1 | |
| *Malus sieversii* | AHJ78581.1 | | KF887980.1 | |
| *Malus toringoides* | AHJ78577.1 | | KF887976.1 | |
| *Manihot esculenta* | cassava4.1_032749m<br>(Phytozome code) | 6 | | |
| *Medicago truncatula* | XP_003622555.1 | 6 | XM_003622507.1 | |
| *Mimulus guttatus* | EYU20441.1 | 6 | KI632284.1 | |
| *Morus notabilis* | XP_010089272.1 | | XM_010090970.1 | |
| *Musa acuminata* | XP_009384463.1 | | XM_009386188.1 | |
| *Nelumbo nucifera* | XP_010264426.1 | | XM_010266124.1 | |
| *Nicotiana tabacum* | AFU10970.1 | | JQ034523.1 | |
| *Nicotiana tomentosiformis* | XP_009592649.1 | | XM_009594354.1 | |
| *Orobanche ramosa* | AEQ30075.1 | | JN412814.1 | |
| *Oryza brachyantha* | XP_006652609.1 | | XM_006652546.1 | |

**Table S2.** *Cont.*

| Species | Protein Code | Intron Number | Gene Bank | Chromosome Number |
|---|---|---|---|---|
| ***Oryza sativa*** | Q7XU29.2 | 6 | | 4 |
| *Panicum virgatum* | Pavir.Gb00928.1 (Phytozome code) | 5 | | |
| *Petunia x hybrida* | ACY01408.1 | | FJ790878.1 | |
| *Phoenix dactylifera* | XP_008796153.1 | | XM_008797931.1 | |
| *Physcomitrella patens* | ADK36680.1 | | HM007802.1 | |
| *Pisum sativum* | ABD67496.2 | | DQ403160.1 | |
| *Populus trichocarpa* | XP_006375244.1 | 6 | XM_006375182.1 | 14 |
| *Prunus mume* | XP_008232618.1 | | XM_008234396.1 | |
| *Prunus persica* | XP_007221108.1 | 5 | XM_007221046.1 | |
| *Pyrus x bretschneideri* | XP_009373202.1 | | XM_009374927.1 | |
| *Ricinus communis* | XP_002511629.1 | 6 | XM_002511583.1 | |
| *Salix purpurea* | SapurV1A.0382s0050.1 (Phytozome code) | 6 | | |
| *Sesamum indicum* | XP_011094476.1 | | XM_011096174.1 | |
| ***Setaria italica*** | XP_004976437.1 | 5 | XM_004976380.1 | 7 |
| *Solanum lycopersicum* | ACY39883.1 | 6 | GQ468556.1 | 1 |
| *Solanum tuberosum* | XP_006359777.1 | 6 | XM_006359715.1 | 1 |
| *Sorghum bicolor* | XP_002446902.1 | 6 | XM_002446857.1 | 6 |
| *Tarenaya hassleriana* | XP_010544591.1 | | XM_010546289.1 | |
| ***Theobroma cacao*** | EOX96379.1 | 6 | CM001879.1 | |
| ***Trifolium repens*** | AHN65153.1 | | KJ127512.1 | |
| *Vitis vinifera* | XP_010648499.1 | 5 | XM_010650197.1 | 15 |
| *Zea mays* | NP_001183928.1 | 6 | NM_001196999.1 | 2 |

In bold, presence of spliced variants.

**Table S3.** CCD8 genes and proteins present in different plant species.

| Species | Protein Code | Intron Number | Gene Bank | Chromosome Number |
|---|---|---|---|---|
| *Actinidia chinensis* | ADP37984.1 | | GU206812.1 | |
| *Aquilegia coerulea* | Aquca_001_00324.1 (Phytozome code) | 5 | | |
| *Arabidopsis lyrata* | XP_002869241.1 | 5 | XM_002869195.1 | |
| *Arabidopsis thaliana* | NP_195007.2 | 5 | NM_119434.3 | 4 |
| *Arabidopsis thaliana* | Q8VY26.1 | | NM_119434.3 | |
| *Arabis alpina* | KFK29890.1 | | CM002875.1 | 7 |
| *Beta vulgaris* | XP_010668385.1 | | XM_010670083.1 | 2 |
| *Boechera stricta* | Bostr.7867s1131.1 (Phytozome code) | 5 | | |
| *Brachypodium distachyon* | XP_003569798.1 | 3 | XM_003569750.2 | |
| *Brassica rapa* | XP_009125426.1 | 5 | XM_009127178.1 | 1 |
| *Camelina sativa* | XP_010432658.1 | | XM_010434356.1 | 10 |
| *Camelina sativa* | XP_010437851.1 | | XM_010439549.1 | 11 |
| *Camelina sativa* | XP_010447340.1 | | XM_010449038.1 | 12 |
| *Camelina sativa* | XP_010447342.1 | | XM_010449040.1 | 12 |
| *Capsella grandiflora* | Cagra.4093s0010.1 | 5 | | |
| *Capsella rubella* | XP_006285526.1 | 5 | XM_006285464.1 | |
| *Carica papaya* | evm.TU.contig_24654.4 | 5 | | |
| *Cicer arietinum* | XP_004501157.1 | | XM_004501100.1 | 5 |
| *Citrus clementina* | XP_006450690.1 | 5 | XM_006450627.1 | |
| *Citrus sinensis* | KDO79823.1 | 5 | KK784877.1 | |
| *Citrus sinensis* | XP_006476130.1 | | XM_006476067.1 | 4 |
| *Crocus sativus* | AIF27229.1 | 5 | KJ361478.1 | |
| *Crocus sativus* | AIF27230.1 | 5 | KJ361479.1 | |
| *Cucumis melo* | NP_001284455.1 | | NM_001297526.1 | |
| *Cucumis melo* | AHY18725.1 | | KJ473491.1 | |
| *Cucumis melo* | XP_008445014.1 | | XM_008446792.1 | |
| *Cucumis sativus* | XP_004148387.1 | 5 | XM_004148339.2 | 2 |
| *Elaeis guineensis* | XP_010917340.1 | | XM_010919038.1 | 1 |
| *Elaeis guineensis* | XP_010924674.1 | | XM_010926372.1 | 6 |
| *Eucaliptus grandis* | KCW81574.1 | | KK198755.1 | |
| ***Eucalyptus grandis*** | XP_010049109.1 | 5 | XM_010050807.1 | |
| *Eucalyptus grandis* | XP_010051229.1 | | XM_010052927.1 | |
| *Eutrema salsugineum* | XP_006412429.1 | 5 | XM_006412366.1 | |
| *Fragaria vesca* | XP_011458989.1 | 4 | XM_011460687.1 | |
| *Glycine max* | XP_003522713.2 | 5 | XM_003522665.2 | 4 |
| *Glycine max* | NP_001242715.1 | 5 | NM_001255786.1 | 6 |
| *Glycine soja* | KHN10194.1 | | KN664752.1 | |
| *Glycine soja* | KHN18168.1 | | KN659929.1 | |
| *Gossypium raimondii* | KJB07492.1 | 5 | CM001740.1 | 1 |
| *Gossypium raimondii* | KJB65669.1 | 5 | CM001749.1 | 10 |
| *Jatropha curcas* | XP_012077359.1 | | XM_012221969.1 | |
| *Linum usitatissimum* | Lus10001599 | 5 | | |
| *Malus baccata* | AIN41153.1 | | KF887984.1 | |
| *Malus baccata* | AIN41152.1 | | KF887983.1 | |
| *Malus domestica* | XP_008352014.1 | 6 | XM_008353792.1 | 15 |
| *Malus domestica* | XP_008361152.1 | 4 | XM_008362930.1 | 15 |
| *Malus domestica* | XP_008378214.1 | 5 | XM_008379992.1 | 8 |
| *Malus hupehensis* | AIN41154.1 | | KF887985.1 | |
| *Malus hupehensis* | AIN41155.1 | | KF927168.1 | |
| *Manihot esculenta* | cassava4.1_005134m | 5 | | |
| *Medicago truncatula* | XP_003603610.1 | 5 | XM_003603562.1 | 3 |
| *Medicago truncatula* | KEH23011.1 | 5 | CM001223.2 | 7 |
| *Mimulus guttatus* | EYU20435.1 | 5 | KI632284.1 | |
| *Musa acuminta* | XP_009389368.1 | | XM_009391093.1 | 2 |
| *Musa acuminta* | XP_009407022.1 | | XM_009408747.1 | 6 |
| *Nelumbo nucifera* | XP_010249576.1 | | XM_010251274.1 | |
| *Nicotiana sylvestris* | XP_009779163.1 | | XM_009780861.1 | |

**Table S3.** *Cont.*

| Species | Protein Code | Intron Number | Gene Bank | Chromosome Number |
|---|---|---|---|---|
| *Nicotiana tabacum* | AGO64766.1 | | KC795555.1 | |
| *Nicotiana tomentosiformis* | XP_009626361.1 | | XM_009628066.1 | |
| *Oryza brachyantha* | XP_006646317.1 | | XM_006646254.1 | 1 |
| *Oryza sativa* | NP_001044229.2 | | NM_001050764.2 | |
| *Oryza sativa* | Q8LIY8.1 | | OSJNBa0014K08.38 | |
| *Panicum virgatum* | Pavir.Ea02892.1 (Phytozome code) | 3 | | |
| *Panicum virgatum* | Pavir.Eb03110.1 (Phytozome code) | 3 | | 5 |
| *Petunia hybrida* | AAW33596.1 | | AY746977.1 | |
| *Phaseolus vulgaris* | XP_007137221.1 | 5 | XM_007137159.1 | 9 |
| *Phoenix dactylifera* | XP_008810664.1 | | XM_008812442.1 | |
| *Phoenix dactylifera* | XP_008804630.1 | | XM_008806408.1 | |
| *Physcomitrella patens* | ADK36681.1 | | HM007803.1 | |
| *Pisum sativum* | AAS66907.1 | | AY557342.1 | 3 |
| *Populus trichocarpa* | XP_002309543.1 | 5 | XM_002309507.1 | |
| *Populus trichocarpa* | XP_002324797.1 | 5 | XM_002324761.1 | |
| *Prunus mume* | XP_008220105.1 | | XM_008221883.1 | |
| *Prunus persica* | XP_007222386.1 | 5 | XM_007222324.1 | |
| *Ricinus communis* | XP_002516503.1 | 4 | XM_002516457.1 | |
| *Salix purpurea* | SapurV1A.0272s0100.1 | 5 | | |
| *Sesamum indicum* | XP_011070469.1 | | XM_011072167.1 | |
| *Sesamum indicum* | XP_011082234.1 | | XM_011083932.1 | |
| *Setaria italica* | XP_004972173.1 | | XM_004972116.1 | |
| *Solanum lycopersicum* | NP_001266276.1 | 5 | NM_001279347.1 | |
| *Solanum tuberosum* | XP_006359761.1 | | XM_006359699.1 | |
| *Sorghum bicolor* | XP_002458477.1 | | XM_002458432.1 | |
| *Tarenaya hassleriana* | XP_010548752.1 | | XM_010550450.1 | |
| *Theobroma cacao* | XP_007012130.1 | 5 | XM_007012068.1 | |
| *Theobroma cacao* | EOY29749.1 | | CM001887.1 | |
| *Triticum urartu* | EMS51709.1 | | KD216346.1 | |
| *Vitis vinifera* | XP_002281239.1 | 4 | XM_002281203.2 | 4 |
| *Zea mays* | NP_001183929.1 | 2 | NM_001197000.1 | 3 |

In bold, presence of spliced variants.
